# The difficulty to model Huntington’s disease in vitro using striatal medium spiny neurons differentiated from human induced pluripotent stem cells

**DOI:** 10.1038/s41598-021-85656-x

**Published:** 2021-03-25

**Authors:** Kim Le Cann, Alec Foerster, Corinna Rösseler, Andelain Erickson, Petra Hautvast, Sebastian Giesselmann, Daniel Pensold, Ingo Kurth, Markus Rothermel, Virginia B. Mattis, Geraldine Zimmer-Bensch, Stephan von Hörsten, Bernd Denecke, Tim Clarner, Jannis Meents, Angelika Lampert

**Affiliations:** 1 Institute of Physiology, RWTH Aachen University, Pauwelsstrasse 30, 52074 Aachen, Germany; 2 Intitute of Human Genetic, RWTH Aachen University, 52074 Aachen, Germany; 3 Institute of Biology II, Division of Functional Epigenetics in the Animal Model, RWTH Aachen University, 52074 Aachen, Germany; 4 Institute Für Biology II, Department Chemosensation, AG Neuromodulation, 52074 Aachen, Germany; 5 Cedars-Sinai Medical Center, Los Angeles, CA 90048 USA; 6 Present Address: Fujifilm Cellular Dynamics, Madison, WI 53711 USA; 7 Intitute of Virology, Clinical and Molecular Virology, Animal Center of Preclinical Experiments (PETZ), 91054 Erlangen, Germany; 8 Aachen – RWTH Aachen, 52074 Aachen, Germany; 9 Intitute for Neuroanatomy, MIT 1, 52074 Aachen, Germany; 10 Present Address: Multi Channel Systems MCS GmbH, Aspenhaustrasse 21, 72770 Reutlingen, Germany

**Keywords:** Cellular neuroscience, Ion channels in the nervous system, Molecular neuroscience, Stem cells in the nervous system

## Abstract

Huntington’s disease (HD) is an autosomal dominant neurodegenerative disorder caused by an expanded polyglutamine repeat in the *huntingtin* gene. The neuropathology of HD is characterized by the decline of a specific neuronal population within the brain, the striatal medium spiny neurons (MSNs). The origins of this extreme vulnerability remain unknown. Human induced pluripotent stem cell (hiPS cell)-derived MSNs represent a powerful tool to study this genetic disease. However, the differentiation protocols published so far show a high heterogeneity of neuronal populations in vitro. Here, we compared two previously published protocols to obtain hiPS cell-derived striatal neurons from both healthy donors and HD patients. Patch-clamp experiments, immunostaining and RT-qPCR were performed to characterize the neurons in culture. While the neurons were mature enough to fire action potentials, a majority failed to express markers typical for MSNs. Voltage-clamp experiments on voltage-gated sodium (Nav) channels revealed a large variability between the two differentiation protocols. Action potential analysis did not reveal changes induced by the HD mutation. This study attempts to demonstrate the current challenges in reproducing data of previously published differentiation protocols and in generating hiPS cell-derived striatal MSNs to model a genetic neurodegenerative disorder in vitro.

## Introduction

Huntington’s disease (HD) is an autosomal dominant neurodegenerative disorder characterized by cognitive deficits, motor impairments and psychiatric symptoms. The mutation responsible for HD is an abnormal expansion of a CAG repeat within exon 1 of the *huntingtin* gene located on chromosome 4 and encoding the huntingtin protein. The CAG nucleotide triplet codes for a polymorphic polyglutamine (polyQ) stretch^1,2^. The non-diseased population carries 9 to 35 polyQ repeats, whereas CAG repeat expansion exceeding 36 repeats leads to HD^2^. The huntingtin protein is expressed ubiquitously but a particular neuronal population in the central nervous system (CNS) is specifically impacted by the mutation: the medium spiny neurons (MSNs), which represent 90 to 97% of the striatal neuronal population^2^. Striatal neurodegeneration usually starts a decade before symptom onset, which usually occurs around the age of 40^3^. The specific vulnerability of the striatal MSNs is still unexplained and there is no treatment to slow the progression of HD, which is always lethal within 15 to 20 years following symptom onset^4^.

Several transgenic and knock-in rodent models have been generated to investigate HD pathological features^5^. They revealed some important mechanisms such as a decreased dendritic spine density^6^ or a down-regulation of the voltage-gated sodium channel (Nav) β4 auxiliary subunit^7^. The Navβ4 subunit is highly expressed in the striatum and in striatal projection fibers. It modulates sodium channel activity and regulates neurite outgrowth^8^. Its expression level is significantly reduced in *post mortem* tissue of HD patients and in three rodent models of HD^7,9,10^. A Navβ4 down regulation in WT mice was shown to affect neurite outgrowth and to decrease repetitive firing frequency in mouse MSNs^7,11^. However, significant species differences between rodent and human cells limit the use of HD rodent models to accurately represent the disease and to predict the electrical activity in human striatal MSNs.

As an alternative, patient-derived human induced pluripotent stem (hiPS) cells have emerged as a powerful tool to decipher mechanisms underlying MSN degeneration and to investigate their firing properties^12^. The reprogramming of skin fibroblasts or mesenchymal stromal cells (MSCs) into human iPS cells allows the generation and the in vitro study of human neurons carrying the huntingtin mutation^4,13^. hiPS cells retain their genetic background and can generate striatal neurons. These essential properties gave rise to the establishment of many hiPS cell-derived MSN differentiation protocols.

To investigate the reliability and reproducibility of these already-existing protocols, we selected and established two previously published protocols^14,15^ to generate striatal MSNs and to investigate their firing activity. We used hiPS cell lines from non-HD donors as well as from different juvenile-onset HD patients and the differentiated neurons were functionally characterized using electrophysiology, immunostaining and quantitative PCR. Our findings highlight the challenges that are innate to the study of hiPS cell-derived MSNs and the difficulties in interpreting data derived from a single differentiation protocol.

## Methods

### Subjects

All patients involved in this study signed an informed consent and the study was approved by the local ethics committees as detailed below (see also Supplementary Table S1). All experiments were conducted in accordance with the Declaration of Helsinki and approved by the local ethics committee of the RWTH Aachen University, Germany.

Healthy Caucasian non-HD subject fibroblasts from Ctrl1 and Ctrl3 were obtained from the healthy donors under their consent^16^ in accordance with the EK 206_09 ethical application approved by local ethics committee of the RWTH Aachen University, Germany^17^.

HD72 human iPS cell line (Cat. Nr. GM23225) was obtained from Coriell Institute for Medical Research (New Jersey, USA) under their consent and privacy guidelines (https://www.coriell.org/).

Healthy human iPS cell Ctrl2 and the HD iPS cell HD109 and HD180 lines were provided by the Cedars-Sinai Medical Center (Los Angeles, California) and informed consent was obtained in accordance with the local ethics committee at the Cedars-Sinai Medical Center (Los Angeles, California, IRB/SCRO protocols Pro00021505 and Pro00024899).

One clone per cell line (six clones in total), corresponding to the biological replicate, with a passage number ranging from 18 to 41, was investigated in this study. One to three technical replicates (the number of differentiations) were performed for each clone (Supplementary Table S4). To be able to perform thorough patch-clamp experiments for all clones and conditions, the differentiations needed to be started at slightly different time points.

### Generation and maintenance of hiPS cells

All cell lines were obtained as hiPS cells and reprogramming of donor cells into hiPS cells was not part of this study (Supplementary Table S2). Human iPS cells were maintained at 37 °C with 95% O_2_—5% CO_2_. They were cultivated either on 0.1% Matrigel hESC-qualified matrix (Becton Dicksinson, 354277) or on 0.1% Geltrex LDEV-Free Reduced Growth Factor Basement Membrane Matrix (Life Technologies, A14133-02). They were supplied daily with either mTeSR 1 supplemented with 5 × mTeSR 1 Supplement or E8 medium supplemented with TeSR—E8 Basal Medium—E8 Supplement (both Stemcell Technologies, Cologne, Germany).

### HD mouse line BACHD

Both heterozygous C57Bl/6 BACHD and homozygous WT C57Bl/6 mice of either sex were used in this study. All methods were carried out in accordance with relevant guidelines and regulations. All experiments were performed according to the approval number 54-2532.1-49/12 by local ethical boards of the District Government of Middle Franconia, Bavaria, Germany.

### Differentiation into MSNs according to the Stanslowsky protocol

**Figure 1:**
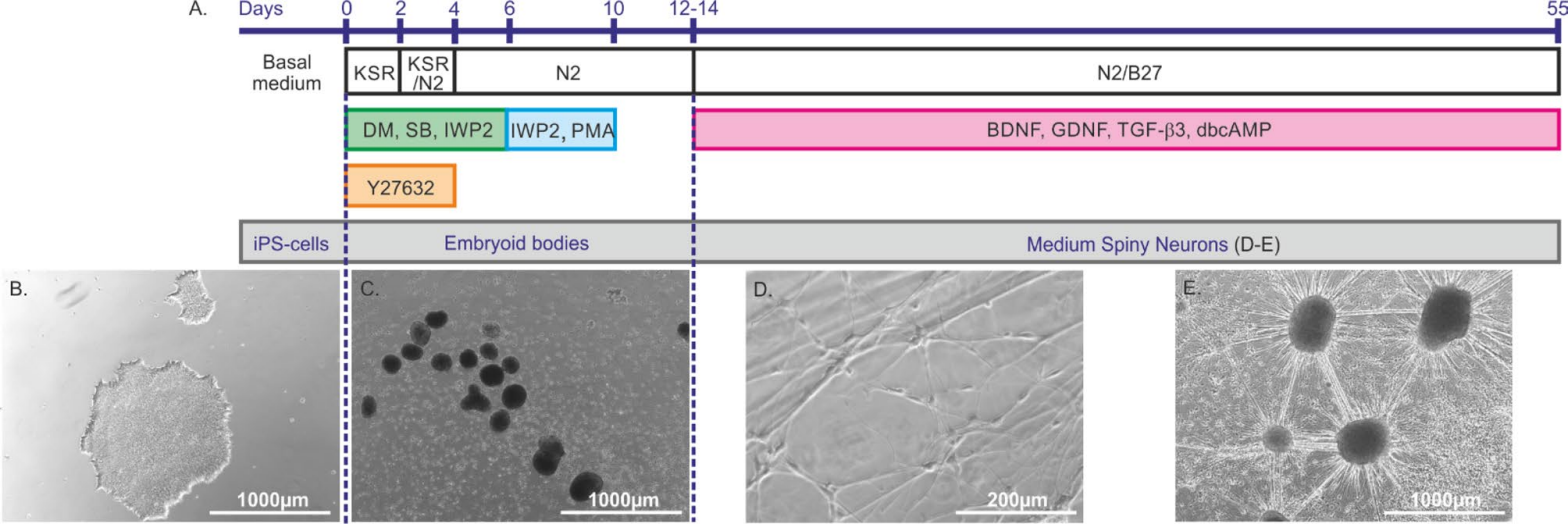
Schematic representation of the Stanslowsky protocol. (**A**) Timeline of the differentiation protocol. DM, SB and IWP2 induce the neural pathway driving the formation of embryoid bodies. IWP2 associated with PMA induces the regional patterning towards the subpallium that comprises large parts of the basal ganglia including striatum and globus pallidus. Y27632 inhibits apoptosis to prevent cell decline at the beginning of the differentiation. The neuronal growth factors BDNF, GDNF and TGF-β3 terminate the differentiation into MSNs and drive their maturation, while dbcAMP increases the intracellular Ca^2+^ levels and contributes to the maturation process. (**B**) hiPS cells grow in basal medium until DIV0. DIV0 to DIV12-14 initiates the formation of embryoid bodies. (**C**) From DIV12-14 to DIV55 ± 3, the neurons are exposed to growth factors to induce neurite outgrowth (**D**) and to establish neuronal networks (**E**). Scale bar: 1,000 µm (**B**, **C**, **E**) and 200 µm (**D**). The dotted lines delineate the different developmental stages during differentiation of MSNs from hiPS cells.

The Stanslowsky protocol^14^ was used to differentiate Ctrl1, Ctrl2, Ctrl3, HD72, HD109 and HD180 hiPS cell lines (Fig. 1).

hiPS cells were cultured daily with 1 mL/well mTeSR1 or E8 medium until they formed colonies (Fig. 1B). Once reaching 70% to 90% confluence, they were split using 0.5 mM EDTA and transferred into ultra-low attachment cell plates (Corning, Life Sciences, USA) containing KSR medium to form embryoid bodies at DIV0 (Fig. 1C). KSR medium was composed of knock-out DMEM supplemented with 20% knock-out serum replacement, 0.1 mM β-mercaptoethanol, 1% 100 × penicillin/streptomycine, 1% 200 mM L-glutamine, and 0.1 mM nonessential amino acids (all Thermo Fisher Scientific, Schwerte, Germany). 5 µM Y-27632 (ROCK, Abcam, Bristol, United Kingdom), 1 µM Dorsomorphin (DM, Tocris Bioscience, Bristol, United Kingdom), 10 µM SB-431542 (SB, Miltenyi Biotec, Bergisch Gladbach, Germany) and 1 µM IWP2 (Selleck Chemicals, Munich, Germany) were added to the KSR medium. Two days later, the medium was replaced with 1:1 mixture of KSR and N2 medium (DMEM/F-12 with 1:100 N2 supplement (Life Technologies) and 1% Penycillin/Streptomycin/L-Glutamine (PSG)) supplemented with the same concentrations of ROCK, DM, SB and IWP2. On day in vitro 4 (DIV4), the medium was replaced with N2 medium using the same combination of small molecules. On DIV6 and DIV8, N2 medium was prepared with 0.6 µM Purmorphamine (PMA, Miltenyi Biotec) and 1 µM IWP2. On DIV10 and DIV12, N2 medium was added without any small molecules. On DIV14, embryoid bodies were transferred onto glass cover slips coated overnight with Matrigel (Sigma-Aldrich, Taufkirchen, Germany) or Geltrex (Life technologies, Waltham, Massachusetts, United States) in N2B27 maturation medium (DMEM/F-12 containing 1:200 N2, 1:100 B27 without vitamin A (Life Technologies) and 1% PSG) supplemented with 20 ng/mL brain-derived neurotrophic factor (BDNF), 10 ng/mL glial-derived neurotrophic factor (GDNF), 1 ng/mL transforming growth factor β3 (TGF-β3) (all PeproTech, Hamburg, Germany) and 0.5 mM di-butyryl cyclic AMP (dbcAMP, Stemcell Technologies). The medium was changed every four days. We decided to extend the maturation time of the differentiated neurons up to DIV55, instead of DIV40 in the initial Stanslowsky protocol (Fig. 1D,E) (see Results).

### Differentiation into MSNs according to the Fjodorova protocol^15^

**Figure 2:**
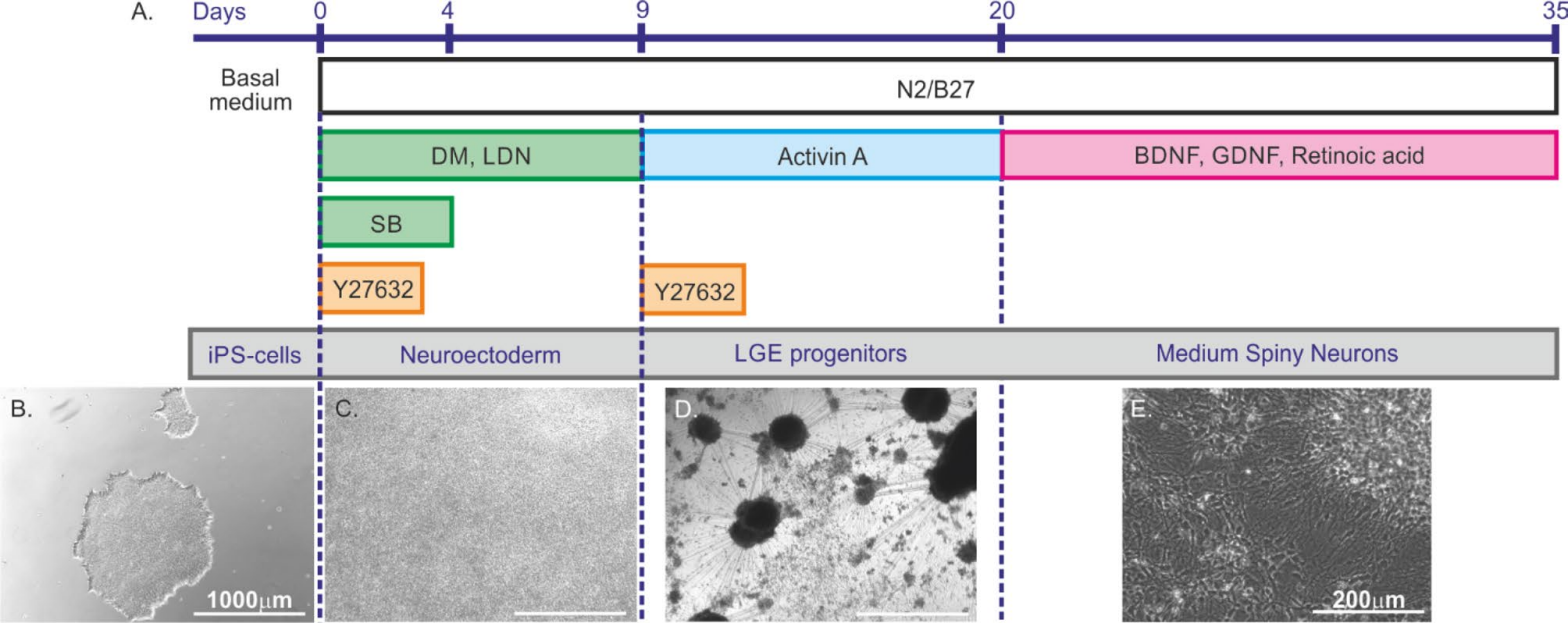
Schematic representation of the Fjodorova protocol. (**A**) Timeline of the differentiation protocol. DM, LDN and SB favor neural induction by inhibiting the dual SMAD signaling. Activin A induces a regional patterning towards the lateral ganglionic eminence that originates from the subpallium. The neuronal growth factors BDNF and GDNF terminate the differentiation into MSNs and mature the neurons. (**B**) hiPS cells grow in basal medium until DIV0. (**C**) Between DIV0 and DIV9, hiPS cells grow into neuroectodermal cells. (**D**) From DIV9 to DIV20, Activin A application induces the formation of lateral ganglionic eminence (LGE) progenitors. (**E**) Between DIV20 and DIV35 ± 3, the LGE progenitors mature under the influence of neuronal growth factors. Scale bar: 1000 µm (**B**, **C**, **D**) and 200 µm (**E**). The dotted lines delineate the different developmental stages during differentiation of MSNs from hiPS cells.

The Fjodorova protocol^15^ was used to differentiate Ctrl1, Ctrl2, HD72, HD109 and HD180 hiPS cell lines (Fig. 2).

hiPS cells were cultured daily with 1 mL/well mTeSR1 or E8 medium until they formed colonies 80% to 90% confluent (Fig. 2B). Cells were split using 0.5 mM EDTA and transferred into wells coated with Geltrex. They were cultivated for 10 days in N2B27 medium (64% DMEM/F-12 + GlutaMAX-1 (Life Technologies), 30% Neurobasal medium (Life technologies), 1% Penicillin/Streptomycin, 1:150 N2 supplement, 1:150 B27 supplement without vitamin A and 0.1% 50 mM β-ME) supplemented with 10 µM SB, 100 nM LDN-193189 (Miltenyi Biotec), 200 nM DM and 10 µM ROCK. Half the medium was replaced every two days. Between DIV5 and DIV9, N2B27 medium was supplemented with 100 nM LDN-193189 (Miltenyi Biotech) and 200 nM DM (Fig. 2C). On DIV9, cells were incubated one hour with N2B27 medium supplemented with 10 µM ROCK and 25 ng/mL Recombinant Human Activin A (Biolegend, San Diego, United States) before being split with 0.02% 0.5 M EDTA and transferred at a 1:10 ratio into wells coated with Geltrex. The N2B27 medium with Activin A was changed every other day until DIV21 (Fig. 2D). At DIV21, cells were split again and 10,000 to 20,000 cells were distributed onto each cover slip of a 24 well plate coated overnight with Geltrex. Cells were maintained in N2B27 medium supplemented with 10 ng/mL BDNF, 10 ng/mL GDNF (all PeproTech) and 100 nM retinoic acid (Sigma-Aldrich). The medium was replaced every four days until DIV35 ± 3 (Fig. 2E). The maturation time was not extended compared to the original publication of this protocol^15^ (see Results).

### Huntingtin gene sequencing

Genomic DNA of hiPS cells was isolated using the NucleoSpin Tissue Kit (Macherey–Nagel, Düren, Germany) according to the manufacturer’s instructions. The primers used to identify the number of CAG repeats within the *huntingtin* gene are available upon request. The number of CAG repeats is indicated in Supplementary Table S3.

### RNA extraction and RT-qPCR

RNA was isolated with the NucleoSpin RNA Kit (Macherey–Nagel) according to the manufacturer’s instructions. RNA was reverse transcribed into cDNA using SensiFAST cDNA Synthesis Kit (Bioline, Luckenwalde, Germany). Quantitative PCR was performed on a RotorGeneQ Real-Time cycler (Qiagen, Hilden, Germany) with SensiMix SYBR No-ROX Kit (Bioline). Data are shown as the relative mRNA expression mean ± 95% confidence interval. mRNA expression of each gene was first normalized to the geometric mean of the mRNA expression of three of the following housekeeping genes: GAPDH, PSMB4, HPRT, B2M and β-actin. The human-specific primers (Eurofins Genomics) are listed in Supplementary Table S7. The data of the hiPS cell-derived neurons of each cell line generated by the two protocols were compared to each other^14,15^.

### Immunofluorescence imaging and quantification

At the end of the differentiation process, the neurons were fixed with 4% paraformaldehyde and blocked and permeabilized with 0.2% bovine serum albumin, 0.1% Triton X-100 in PBS and 3% normal goat serum (all Sigma-Aldrich). Neurons were stained with mouse anti-βIII tubulin (or clone Tuj1, MAB1195, Abcam, R&D Systems, 1:400), rabbit anti-βIII tubulin (Sigma-Aldrich, 1:1000), mouse anti-GAD67 (clone 1G10.2, Merck Millipore, Darmstadt, Germany, 1:200), rabbit anti-DARPP32 (Abcam, 1:100), rabbit anti-SCN4B (Abcam, 1:500), rabbit anti-DRD1 (Abcam, 1:200), rabbit anti-TH (ab152Merck Millipore, Darmstadt, Germany, 1:500), mouse anti-peripherin (Santa Cruz, Heidelberg, Germany, 1:100). The presence of striatal interneurons was investigated using the following primary antibodies: rabbit anti-neuropeptide Y (Immunostar 22,940, Wisconsin, United States, 1:500), rat anti-somatostatin (Merck Millipore MAB354, 1:500), rabbit anti-somatostatin (T4103Dianova, Hamburg, Germany,, 1:500), mouse anti-calretinin (Swant 6B3, Switzerland, 1:500), rabbit anti-neuropeptide VIP (Immunostar 20077, 1:500), rabbit anti-parvalbumin (Swant, 1:1,000). The secondary antibodies used were goat anti-mouse IgG Alexa Fluor 488, goat anti-rabbit IgG Alexa Fluor 594, goat anti-rabbit IgG Alexa Fluor 488 (both Life technologies, 1:1,000), goat anti-rabbit Cy3 (Jackson, Cambridge, United Kingdom, 1:2,000), goat anti-rat Cy3 (Invitrogen, Schwerte, Germany, 1:2,000), goat anti-mouse Cy3 (Jackson, 1:2,000). Nuclei were counterstained with DAPI (Thermo Fisher Scientific). Three to five random regions of interest (ROI) were investigated for each cover slip using an LSM700 confocal microscope (Carl Zeiss, Oberkochen, Germany) with 40 × oil immersion objective or a DMi8 inverted microscope with instant computational clearing (Leica, Germany) with 20 × objective. Counting was performed manually using the ImageJ-win64 software and the observer was blinded to the cell type and to the protocols. To detect nucleus staining in dense culture condition, the contrast brightness/background was increased. We counted as neurons only cells with a nucleus fully embedded by a fluorescence signal that suggested a close interaction with the cell membrane. Results of ROI counting were averaged and presented as mean.

### Electrophysiology

Whole-cell patch-clamp experiments were performed with an EPC-10 USB amplifier (HEKA Elektronik, Lambrecht, Germany) at room temperature (20–22 °C). Glass pipettes of 2–4 MΩ (Biomedical Instruments) were manufactured with a DMZ puller (Zeitz Instruments GmbH, Martinsried, Germany). Sampling rate was set to 50 kHz for the recordings while using a 10 kHz low-pass filter. Series resistance (< 5.5MΩ for voltage-clamp mode) was compensated by at least 70%. Leak currents were subtracted online using the P/4 procedure. The liquid junction potential was not corrected.

### Voltage-clamp recordings

The following external solution was used (in mM): 140 NaCl, 1 MgCl_2_, 1 CaCl_2_, 10 HEPES, 1 glucose, 20 TEA-Cl, 1 4-AP, 0.1 CdCl_2._ (pH7.3; 310–320 mOsm). Some neurons were recorded with 500 nM Tetrodotoxin (TTX, Tocris Bioscience) diluted in external solution and applied through a gravity-driven perfusion system. Two intracellular solutions were used, with and without 500 nM intracellular free Ca^2+^. The solution without Ca^2+^ contained (in mM): 10 NaCl, 140 CsF, 1 EGTA, 10 HEPES, 5 glucose and 5 TEA-Cl. The solution with Ca^2+^ contained (in mM): 10 NaCl; 140 CsCl; 1 EGTA; 10 HEPES; 5 glucose, 0.82 Ca^2+^ (both solutions pH7.3; 300-315 mOsm).

For all voltage-clamp protocols, the holding potential was set to − 120 mV. Voltage dependence of activation was assessed from holding potential using 40 ms pulses to a range of test potentials from − 100 mV to + 60 mV in 10 mV increment (test-pulse) with an interval of 5 s. To isolate somatic currents and avoid space clamp artefacts, a voltage pre-pulse protocol was used to inactivate distant sodium channels^16,20^ (Fig. 6A). The pre-pulse (− 60 mV to − 20 mV for 4 or 5 ms) was followed by a repolarizing inter-pulse (− 120 to − 70 mV for 1 ms) which preceded the regular test-pulse. In few cells no pre-pulse was applied due to small current amplitude and because sodium currents were well clamped without pre-pulse. Pre-pulse and inter-pulse voltage and duration were adjusted to each cell individually to obtain optimal voltage-clamp conditions. Subsequently, 500 nM TTX was applied in a limited number of cells to measure the TTX-sensitive current. Conductance (*G*) was calculated at each voltage (*V*) using the equation $G=\frac{\mathrm{I}}{\mathrm{V}-\mathrm{Vrev}}$ with *V*_*rev*_ being the reversal potential and *I* being the inward current at the respective voltage. Conductance-voltage curves were fit using a Boltzmann equation $\frac{G}{Gmax}=Gmin+\frac{Gmax-Gmin}{1+exp\frac{\mathrm{Vhalf}-\mathrm{Vm}}{\mathrm{k}}}$ where *G*_*min*_ and *G*_*max*_ are the minimum and maximum of sodium conductance respectively, V_half_ is the potential of half-maximal channel activation and *k* is the slope factor.

Steady-state fast inactivation was measured with or without 500 nM intracellular Ca^2+^ concentration using a two-step protocol. A 500 ms pre-pulse with potentials ranging from − 120 mV to + 10 mV in 10 mV incremental steps was used to inactivate Nav channels. This pre-pulse was immediately followed by a 40 ms test-pulse to 0 mV, allowing the determination of the remaining fraction of available channels. Relative current was calculated as test-pulse current at each voltage (*Vm*) divided by the maximum test-pulse current and plotted against the pre-pulse voltage. Relative currents were fitted with the above Boltzmann equation.

### Current-clamp recordings

Extracellular bath solution contained (in mM): 140 NaCl; 3 KCl; 1 MgCl_2_; 1 CaCl_2_; 10 HEPES; 20 glucose (pH 7.3; 290–310 mOsm). As for voltage-clamp recordings, two intracellular solutions were used, with or without 500 nM free [Ca^2+^]_i_. The exact concentration of free Ca^2+^ was calculated using the Maxchelator website (Temperature 20 °C; pH 7.3)^18^. The intracellular solution without Ca^2+^ contained (in mM): 4 NaCl, 135 K-gluconate, 3 MgCl_2_, 5 EGTA, 5 HEPES, 2 Na_2_GTP, 0.3 NaGTP. The intracellular solution with 500 nM intracellular free Ca^2+^ contained (in mM): 4 NaCl, 135 K-gluconate, 3 MgCl_2_, 5 EGTA, 5 HEPES, 2 Na_2_GTP, 0.3 NaGTP, 0.82 Ca^2+^ (pH 7.25; 280–300 mOsm).

The resting membrane potential (RMP) was measured immediately after establishing the whole-cell configuration. Holding current was then adjusted to achieve a membrane voltage of − 70 mV ± 10 mV. Action potentials (APs) were induced by incremental current injections (from 1 to 50 pA in 200 ms) to determine the rheobase, i.e. the amount of current required to trigger one AP. A subsequent protocol with 0.5 × rheobase current injection was made during 200 ms to generate more APs (up to 4.5 × rheobase stimulus). APs were only considered if they exceeded 0 mV. The first AP evoked by the square pulse protocol was used to calculate AP properties. The AP threshold was defined as the minimum of the first derivative of the AP (i.e. the point of inflection during the depolarization). The amplitude was measured between holding voltage and AP peak. The afterhyperpolarization is the minimum voltage following the AP peak. The time-to-peak is the duration between current pulse onset and AP peak. Current-clamp recordings were performed at DIV55 ± 3 (Stanslowsky protocol) and at DIV35 ± 3 (Fjodorova protocol). Neurons that fired two or more APs to a given stimulus were considered tonically firing in our experiments. Neurons firing a single AP were considered as phasic firing.

### Statistical analysis

Data were analyzed using GraphPad Prism version 5 or 6 (GraphPad Software, Inc) and SPSS (IBM SPSS Statistics Version 25). Two groups were compared using a 2-tailed Student’s *t* test or a Mann–Whitney test, depending on normal distribution. Comparison between 3 or more groups was performed using a 1-way ANOVA followed by a Bonferroni, a Tukey’s multiple comparison test or a Kruskall-Wallis test. The exact value of n (number of cells) is indicated in the Fig. legends or in the Tables. Data are presented as mean and error bars denote 95% confidence interval (CI). Outliers were identified and addressed.

## Results

### A longer time in culture increases the percentage of active neurons in the Stanslowsky protocol

**Figure 3:**
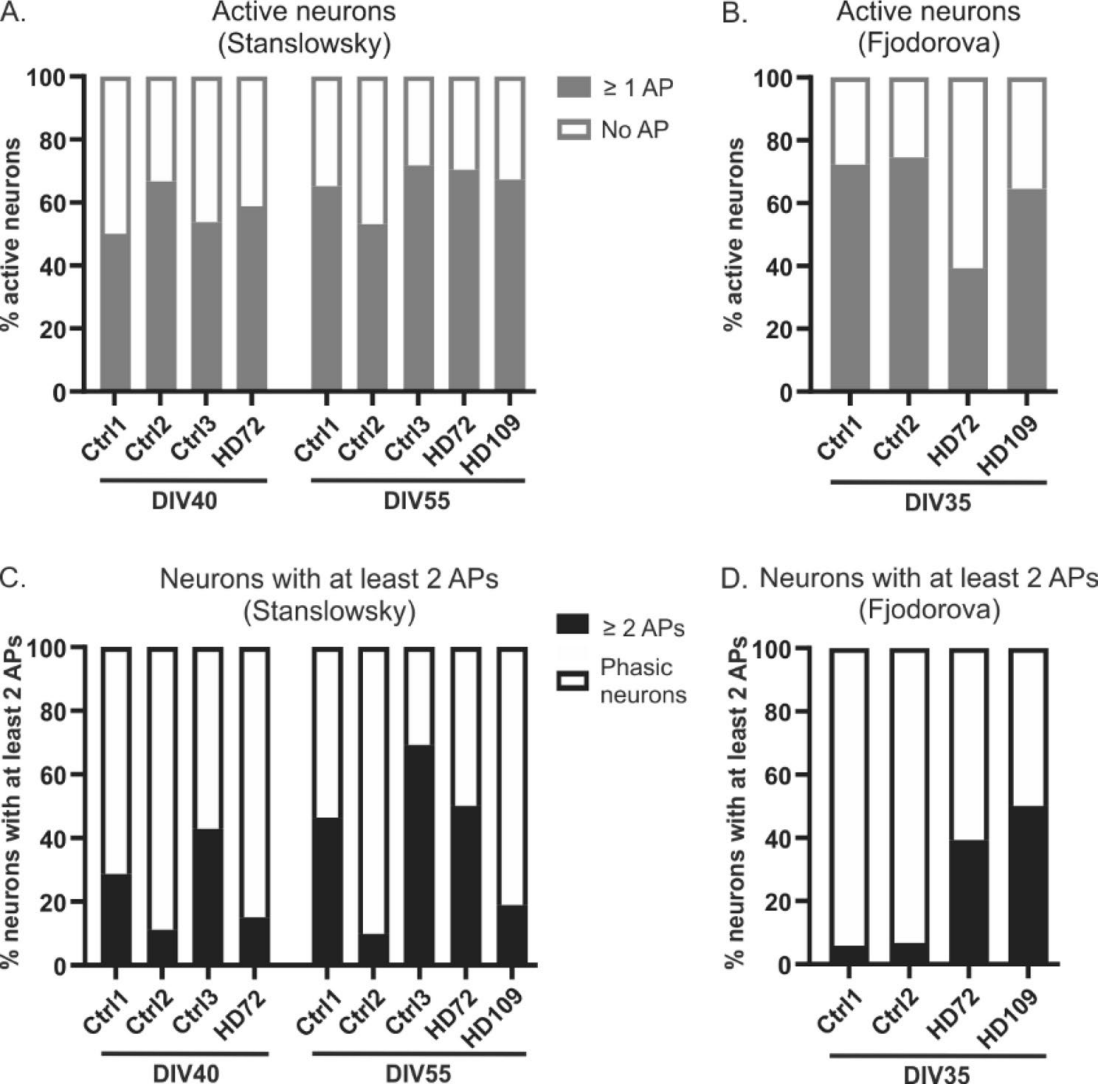
The percentage of active neurons and neurons firing more than 2 APs is increased following a longer time in culture for the Stanslowsky protocol. (**A**) The percentage of active control and HD neurons firing at least one AP at DIV40 ± 2 (n = 19 Ctrl1, 18 Ctrl2, 6 Ctrl3 and 20 HD72 neurons) and the same neurons with HD109 at DIV55 ± 3 (n = 60 Ctrl1, 51 Ctrl2, 28 Ctrl3, 38 HD72 and 37 HD109 neurons) for the Stanslowsky protocol. (**B**) The percentage of active control neurons firing at least 1 AP at DIV35 for the Fjodorova protocol (n = 34 Ctrl1, 73 Ctrl2, 49 HD72 and 20 HD109 neurons). (**C**) The percentage of phasic firing neurons (generating only 1 AP) and of tonic firing neurons (generating two and more APs) at DIV40 and DIV55 for the Stanslowsky protocol. (**D**) The percentage of phasic firing and of tonic firing neurons at DIV35 for the Fjodorova protocol. There was no extended time in culture for the hiPS cell-derived neurons of this protocol. See Supplementary Table S5 for details.

To compare some of the previously published protocols for MSN differentiation from hiPS cells, we selected two well established protocols^14,15^ in order to generate striatal neurons and to characterize their neuronal identity and functional activity. For the latter, it is important to work with neurons that express sufficient amounts of voltage-gated ion channels to be electrically mature. To address this question and prior to any experiment, we aimed to determine the differentiation time in vitro required to reach this functional maturation. Using the Stanslowsky protocol, we found in two control cell lines (Ctrl1 and Ctrl3) and in one HD cell line (HD72) a higher percentage of electrically active neurons (i.e. = neurons firing at least one action potential) at DIV55 than at DIV40 (50%, 53.8% and 58.8% for Ctrl1, Ctrl3 and HD72 respectively at DIV40 vs 65.2%, 71.8% and 70.4% respectively at DIV55) (Fig. 3A). However, this observation was not verified for Ctrl2, with a higher percentage of electrically active neurons at DIV40 (66.7%) compared to DIV55 (53.1%). In addition, the Ctrl1, Ctrl3 and HD72 neurons showed a tendency to fire a higher number of APs following two additional weeks in culture (15 to 42.9% neurons at DIV40 vs 46.4 to 69.2% neurons at DIV55), here again except for Ctrl2 with nearly 10% neurons firing more than 2 APs at DIV40 and DIV55 (Fig. 3C). All together, we decided to extend the maturation time of the differentiated neurons up to DIV55. The control neurons of the Fjodorova protocol displayed a similar electrical activity at DIV35 (72.3 to 100% electrically active neurons) as the neurons of the Stanslowsky protocol at DIV55 and they showed a varying range of tonic firing neurons going from 5.9% to 50% for Ctrl1 and HD109 respectively. Their time in culture was maintained at 35 days (Fig. 3C), in accordance with the initially published protocol^15^.

### Both differentiation protocols produce low amount of GABAergic positive neurons and MSNs

**Figure 4:**
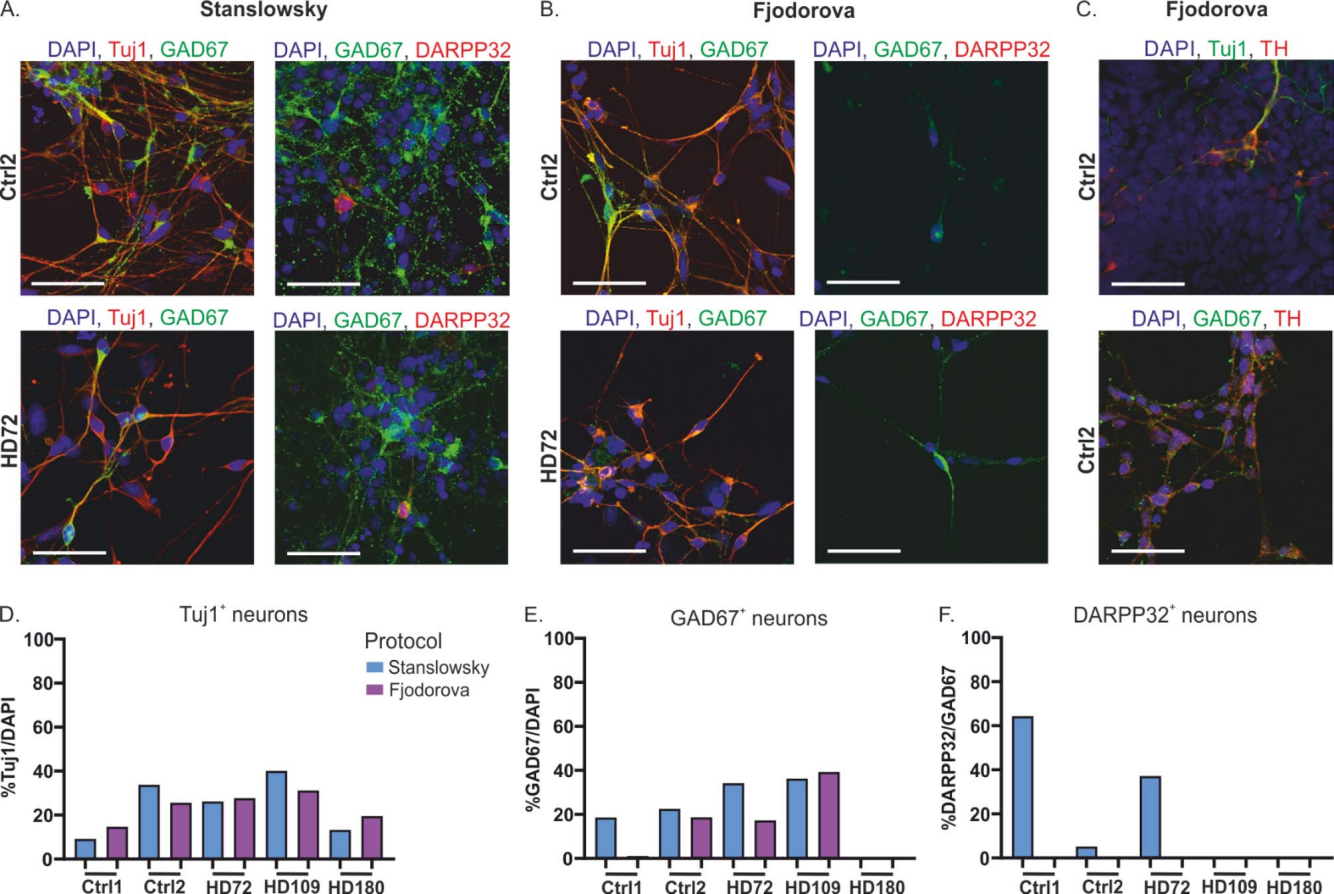
Both differentiation protocols produce only a small percentage of MSNs. (**A**,**B**) Representative immunostaining of Ctrl2 (bottom) and HD72 neurons (top) from the Stanslowsky protocol (**A**) and the Fjodorova protocol (**B**). DAPI staining is represented in blue, Tuj1 and DARPP32 in red and GAD67 in green. Scale: 50 µm. (**C**) Ctrl2 neurons of the Fjodorova protocol stained with Tuj1 in green and TH in red (top) or with GAD67 in green and TH in red (bottom). (**D**) Percentage of Tuj1^+^ neurons in each culture compared to the number of DAPI-stained nuclei. (**E**) Percentage of GAD67^+^ neurons (GABAergic neurons) in each culture compared to the total number of DAPI-stained nuclei. (**F**) Percentage of DARPP32^+^ neurons (striatal MSNs) in each culture compared to the number of GAD67^+^ neurons. See Supplementary Table S6 for quantitative data. Data show mean (1 technical replicate).

Immunostaining was performed on hiPS cell-derived neurons of both the Stanslowsky protocol at DIV55 and Fjodorova protocol at DIV35^14,15^ to characterize their neuronal identity (Fig. 4). We used the neuronal marker Tuj1 to distinguish neuronal and non-neuronal cells (Fig. 4A,B,D). Our cultures were composed of 9.2% to 40.1% Tuj1^+^ neurons of the Stanslowsky protocol and of 14.7 to 31.2% Tuj1^+^ neurons of the Fjodorova protocol (Fig. 4D, Supplementary Table S6). Staining for peripherin, a marker of peripheral neurons, was negative (Supplementary Fig. S1). We are confident that those Tuj1^+^ cells are central neurons.

Striatal MSNs in vivo are all GABAergic^3^ and express the specific marker DARPP32^19^. These neurons provided a large population of GABAergic neurons, with a range of 75% to 81% Tuj1^+^ neurons expressing the enzyme GAD67 (precursor of GABA) in the two cell lines Ctrl2 and HD72 investigated in both protocols (Fig. 4A,B and Supplementary Table S6). Except for Ctrl1 neurons in the Fjodorova protocol and HD180 neurons in both protocols, all hiPS cell-derived neurons expressed GAD67 (Fig. 4A,B,E). In contrast, very few GABAergic neurons expressed the typical MSN marker DARPP32, with a high variability for the neurons of the Stanslowsky protocol, ranging from 0% for the HD109 neurons to 64.4% for the Ctrl1 neurons (Fig. 4A, F and Supplementary Table S6). Results were more disappointing for the Fjodorova protocol without any neuron expressing the DARPP32 marker (Fig. 4B,F and Supplementary Table S6). In summary, both protocols seem to produce only very few striatal MSNs.

As MSNs seemed to be the minority of the cells derived by the differentiation protocols in our hands, we investigated which other neuronal populations were present in our preparations. We stained the neurons with DRD1, a dopaminergic marker expressed by MSNs and other neuronal populations in the brain^20^. 50% to 77% of the Tuj1^+^ neurons investigated expressed DRD1 (Supplementary Fig. S1). We also investigated the expression of tyrosine hydroxylase (TH), the rate-limiting enzyme in the synthesis of catecholamines, among which dopamine. Previous findings revealed that TH is not only found in dopaminergic neurons, but also in striatal interneurons of humans^21,22^ and rodents^23,24^, although not accompanied by dopamine release^24^. In the striatum, these interneurons make GABAergic synapses onto MSNs^24^. We confirmed first the expression of TH in two Tuj1^+^ cell lines where it was investigated: Ctrl2 and HD109 neurons (Fig. 4C, top and Supplementary Table S6). In addition, we revealed the presence of striatal interneurons in our culture, confirmed by a positive staining for somatostatin and calretinin (Supplementary Fig. S1D-F, Table S6). We also tested for the interneuron markers parvalbumin, neuropeptide Y and neuropeptide VIP, but could not identify any stained cells (Supplementary Fig. S1D-F, Table S6). Finally, we investigated whether some of our TH^+^ neurons may also be striatal interneurons. Three out of four striatal interneuron populations are GABAergic^24^, therefore we stained our neurons with TH and GAD67. The two clones Ctrl2 and HD72 revealed a range of 28.5% to 81.5% of TH/GAD67 co-expression (Fig. 4C, Supplementary Table S6).

All together, these findings suggest that the GABAergic neurons in our cultures are characterized by higher striatal interneuron populations than MSNs. Thus, both protocols designed to result in differentiation of MSNs mainly produced central neurons with a mixed identity. Importantly, we found a range of 4.5% to 12.4% of GABAergic calretinin interneurons while a considerable fraction of differentiated neurons are not striatal MSNs, and might therefore be unsuitable for HD-related investigations. Nevertheless, we aimed to investigate their electrophysiological properties, as the strong genetic modification in our HD cell lines could potentially also show its effect in central neurons other than MSNs.

### The Navβ4 subunit is very little expressed in the differentiated neurons of both genotypes

**Figure 5:**
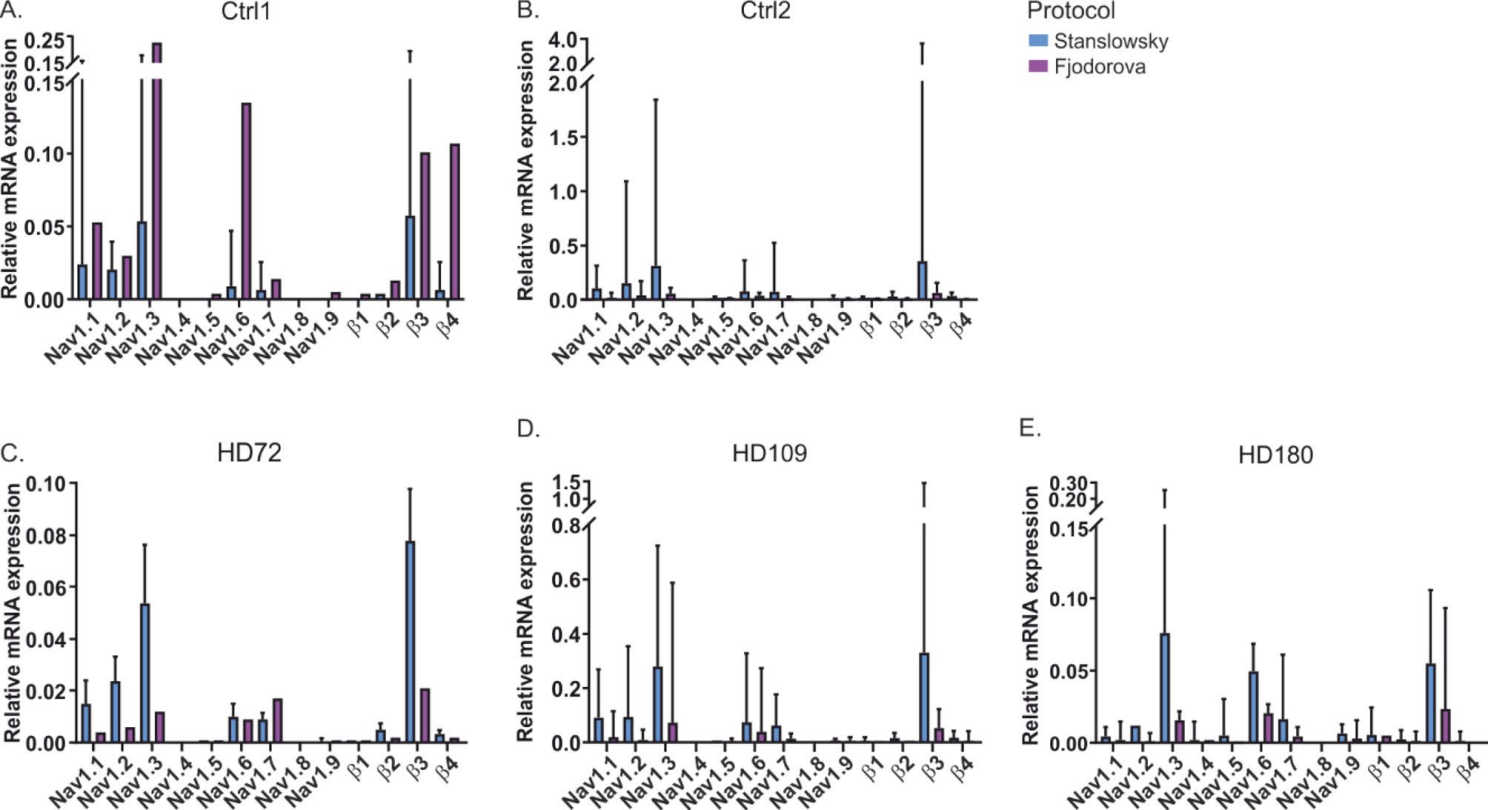
RT-qPCR of the nine Nav α and the four Nav β subunits from each iPSC-derived neuron lines of the Stanslowsky (blue) or the Fjodorova (purple) protocol. (**A**-**B**) RT-qPCR of Ctrl1 (**A**) and Ctrl2 (**B**) neurons. (**C**-**E**) RT-qPCR of HD72 (**C**), HD109 (**D**) and HD180 (**E**) neurons. Error bars denote 95% confidence interval. Number of differentiations for the Stanslowsky protocol: n = 2 for Ctrl1, n = 2 for Ctrl2, n = 3 or HD72, n = 2 for HD109, n = 2 for HD180 and for the Fjodorova protocol: n = 1 for Ctrl1, n = 3 for Ctrl2, n = 1 or HD72, n = 2 for HD109, n = 2 for HD180.

Nav channels are crucial for neuronal function and AP generation. In search for a biomarker of MSN degeneration, the Navβ4 subunit has been reported to be down-regulated in neurons of HD patients and rodent models^7,9^. Therefore, we performed RT-qPCR to investigate expression of Nav α and β subunits in our hiPS cell-derived neurons (Fig. 5).

In general, almost every cell line revealed a higher expression level of all Nav α and β subunits in the neurons differentiated using the Stanslowsky protocol compared to those differentiated using the Fjodorova protocol. We found that all hiPS cell-derived MSN lines expressed Nav1.1, Nav1.2, Nav1.3, Nav1.6 and Nav1.7 as well as the Navβ3 subunit, although expression varied between cell lines and genotype (Fig. 5), probably due to the various number of differentiations performed for each cell line (n = 1–3 differentiations). Navβ1, Navβ2 and Navβ4 are expressed to a lower extent compared to Navβ3 (Fig. 5B,D). These results are in accordance with data from the original Stanslowsky protocol^14^. With regards to the expression of Navα subunits, we found a similar expression pattern in WT mouse striatum (Supplementary Fig. S2). However, we found considerably higher amounts of β-subunit mRNA expression in mouse tissue compared to our human iPS cell-derived neurons. This is interesting in the context that an HD-related down-regulation of the Navβ4 subunit has mostly been based on rodent tissue^7,9^. In fact, we confirmed the Navβ4 subunit down-regulation in BACHD mice, one of the most physiological mouse models^5,6^, using in situ hybridization and RT-qPCR (Supplementary Fig. S2). It is not clear, however, whether this down-regulation also affects human MSNs of HD patients. We were therefore particularly interested in the expression of the Navβ4 subunit in our iPS cell-derived neurons. Importantly, expression of Navβ4 mRNA was found to be very low in all clones, regardless of genotype or differentiation protocol (Fig. 5A-E). These findings question the relevance of the Navβ4 subunit as a biomarker for HD.

### Sodium channel gating is reversely affected by HD genotype in the two differentiation paradigms

**Figure 6:**
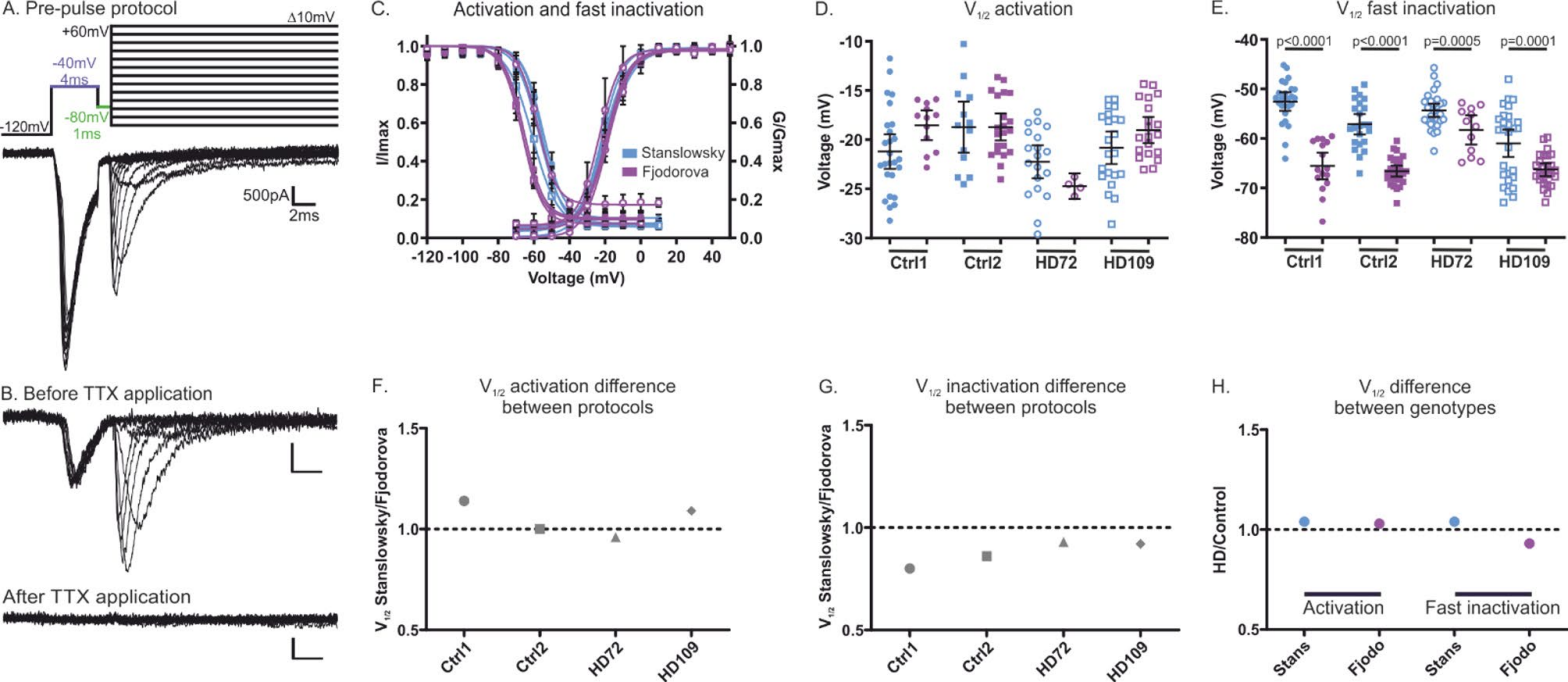
The two protocols reveal large differences in the gating properties of Nav channels expressed in the differentiated neurons. (**A**) Pre-pulse protocol (top) designed to accurately record voltage dependence of activation in mature hiPS cell-derived neurons without space clamp artefacts. Pre-pulse (purple) and inter-pulse (green) are adjusted to each neuron individually. Example trace (bottom) is from Ctrl2 neurons of the Fjodorova protocol. (**B**) Representative recording of HD72 hiPS cell-derived central neurons of the Stanslowsky protocol before 500 nM TTX application (top) and following 500 nM TTX application (bottom). Scale bars are the same as in (**A**). (**C**) Voltage dependence of activation and steady-state fast inactivation of control and HD neurons of the Stanslowsky protocol (blue) and of the Fjodorova protocol (purple) (see Table S8 for results of each individual cell line). (**D**-**E**) Values for half-maximal (V_half_) voltage-dependent activation (**D**) and steady-state fast inactivation (**E**) of Ctrl1 (filled circles, n = 24–26), Ctrl2 (filled squares, n = 13–23), HD72 (open circles, n = 19–30) and HD109 neurons (open squares, n = 20–22) for the Stanslowsky protocol and Ctrl1 (filled circles, n = 12–16), Ctrl2 (filled squares, n = 21–27), HD72 (open circles, n = 12–30) and HD109 neurons (open squares, n = 20–26) for the Fjodorova protocol. Data show mean ± 95% CI. (**F**–**H**) Plot displays the difference between V_half_ values of voltage dependence of activation (**D**) and steady-state fast inactivation (**E**) obtained from the two differentiation protocols when control/HD neurons of each protocol are pooled. A value of 1 (dotted line) represents equal V_half_ values in both protocols. Values < 1 mean hyperpolarized values in the Fjodorova protocol as compared to the Stanslowsky protocol. While both protocols provide comparable values for voltage dependence of activation (**F**), neurons from the Fjodorova protocol consistently display a hyperpolarized fast inactivation (**G**). (**H**) Plot displays the difference between V_half_ values of voltage dependence of activation and steady-state fast inactivation obtained from the two genotypes for each protocol. The genotype does not seem to influence the Nav channel gating of hiPS cell-derived neurons.

Whole-cell voltage-clamp recordings were performed in differentiated neurons to assess whether two different protocols may influence Nav gating in control and HD neurons. A pre-pulse voltage protocol was used to measure whole-cell current in control and HD neurons (Fig. 6A). This protocol allows for good voltage clamp even in cells with long neurites and larger currents^16,25^. 500 nM TTX was applied in a limited number of cells to test whether the TTX-resistant cardiac Nav1.5 channel or the peripheral sensory nerve isoforms Nav1.8 and Nav1.9 are expressed in the differentiated neurons. No TTX-resistant current could be measured in the hiPS cell-derived HD72 neurons (Fig. 6B) and we assumed that all the Nav isoforms expressed in our neurons are TTX-sensitive. These voltage-clamp results are well in line with the RT-qPCR results (Fig. 5), suggesting that the cells revealed by the differentiation protocols are indeed of CNS lineage.

The iPS cell-derived neurons from each protocol showed a similar current density (Supplementary Table S8). Concerning the voltage dependence of activation, a large variability in the half maximal voltage was observed between the neurons of each protocol, and no significant difference was found. This suggests that the protocols do not exert any obvious effect on the activation of the Navs expressed in the differentiated neurons (Fig. 6C-D and Table S8). We found a more depolarized half maximal voltage in all four differentiated neurons from the Stanslowsky protocol (V_half_ = − 52.5 mV (CI − 54.4 to − 50.7 mV) for the Ctrl1 to − 61.0 mV (CI − 63.7 to − 58.2 mV) for the HD109 neurons) compared to those of the Fjodorova protocol (V_half_ = − 58.3 mV (CI − 61.2 to − 55.3 mV) for the HD72 neurons to − 66.6 mV (CI − 67.5 to − 65.5 mV) for the Ctrl2) (Fig. 6C,E and Table S8).

The plots displaying the differences between V_half_ values of activation (Fig. 6F) and V_half_ of fast inactivation (Fig. 6G) sum up the discrepancy within the two protocols observed in the previous panels of Fig. 6, while they do not suggest any obvious effect of the HD genotype on the Nav function of the neurons differentiated in both protocols (Fig. 6H).

All together, these results again highlight the considerable variability induced by the differentiation paradigm. Surprisingly, the largest effects on Nav channel gating do not come from the genotype but from the protocols used to generate hiPS cell-derived striatal neurons. This suggests the presence of neurons with various functions and point out a striking lack of homogeneity within the differentiated neurons derived from the two differentiation protocols. Finally, we did not observe a clear effect of the HD mutation on Nav channel activation and fast inactivation.

### Action potential features are unaffected by HD genotype

**Figure 7:**
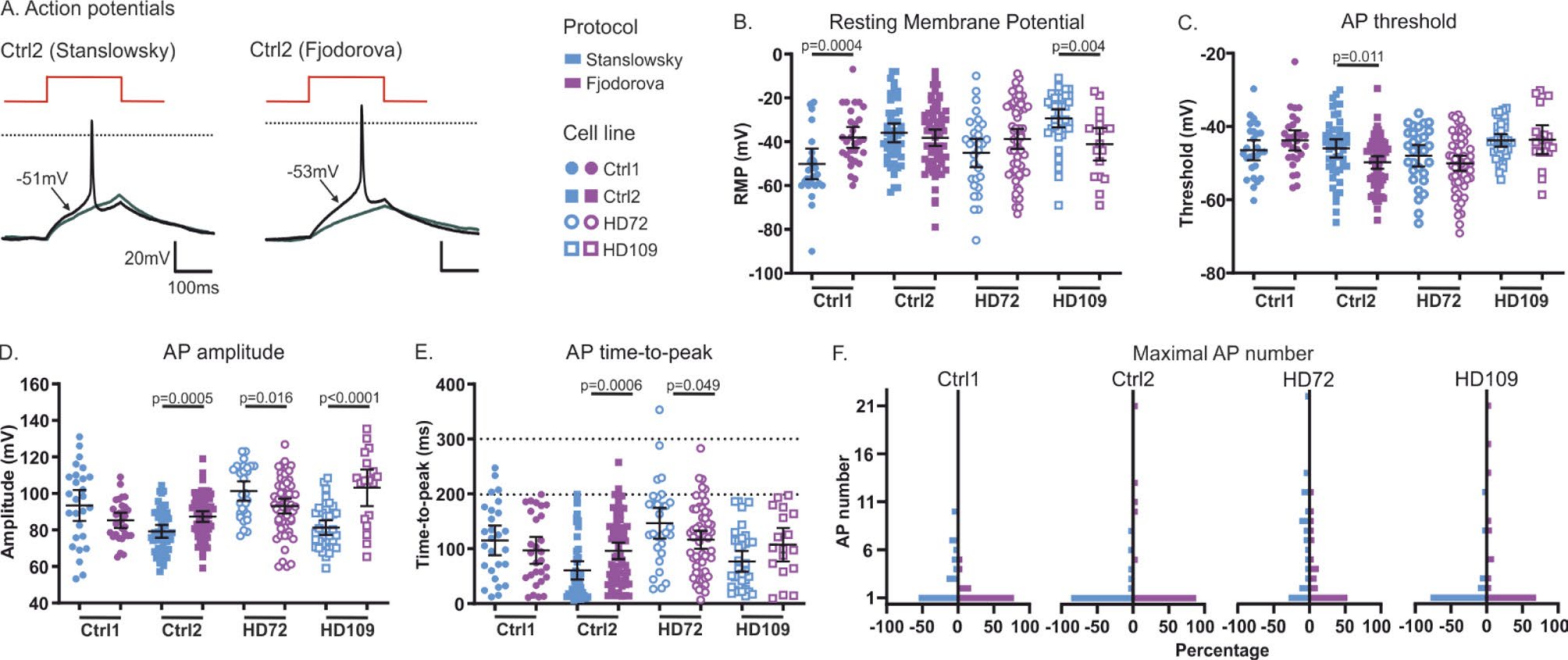
hiPS cell-derived neurons display distinct AP features within the two protocols. (**A**) Representative AP traces (black) of Ctrl2 neurons from Stanslowsky (left) and from Fjodorova (right) protocols. The grey trace represents the subthreshold current injection step. AP threshold for each individual neuron is indicated with an arrow. The red trace indicates the current injection step. Dotted black lines represent 0 mV. (**B**) Resting membrane potential (RMP). (**C**,**E**) AP features of control/HD hiPS cell-derived neurons from the Stanslowsky protocol (blue, n = 27 Ctrl1, 46 Ctrl2, 30 HD72 and 37 HD109 neurons) and the Fjodorova protocol (purple, n = 29 Ctrl1, 65 Ctrl2, 57 HD72 and 18 HD109 neurons): AP threshold (**C**), AP amplitude (**D**) and AP time-to-peak (**E**). Two dotted lines indicate the cut-off of neurons with a time-to-peak longer than 200 or 300 ms. (**F**) Maximal number of APs fired by control and HD neurons. Data show mean ± 95% CI. See Supplementary Table S9 for statistical results.

We previously showed a variability between protocols concerning the fast inactivation (Fig. 6E,F). These significant effects may lead to an alteration of AP features in hiPS cell-derived neurons. We therefore performed current-clamp experiments to determine whether the differentiation protocol may have an effect on their electrical activity (Fig. 7A). The resting membrane potential (RMP) was around − 40 mV (Fig. 7B), which is within ± 5 mV to what has previously been reported^14,26^. Although the RMP was similar for the Ctrl2 and HD72 neurons of both protocols, we found a 12 mV difference more *hyper*polarized RMP for the Ctrl1 neurons of the Stanslowsky protocol (− 50.1 mV (CI − 57.1 to − 43.1 mV)) compared to the same neurons differentiated with the Fjodorova protocol (− 38.1 mV (CI − 42.9 to − 33.3)) (Fig. 7B, Table S9). The opposite was observed for the HD109 neurons, with a 12 mV more *de*polarized RMP for the neurons of the Stanslowsky (− 29.3 mV (CI − 33.5 to − 25.2 mV)) compared to the neurons of the Fjodoroa protocols (− 41.1 mV (CI − 48.5 to − 33.7 mV)). These values again hint at important inconsistencies between differentiation protocols. The AP threshold, amplitude and time-to-peak also showed heterogeneous and contradictive results between the neurons of each protocol (Fig. 7C-E). If the neurons of the three neuronal cell lines Ctrl2, HD72 and HD109 show similar AP threshold values between the two protocols (Fig. 7C), the results vary concerning other AP features. The AP amplitude of Ctrl2 and HD109 neurons is lower in the Stanslowsky protocol (79.3 mV (CI 75.7 to 82.8 mV) for Ctrl2 and 81.3 mV (CI 77.2 to 85.3 mV) for HD72) than in the Fjodorova protocol (87.3 mV (CI 84.4 to 90.2 mV) for Ctrl2 and 87.3 mV (CI 83.8 to 90.7 mV) for HD72) while the contrary is found with the HD72 neurons (101.3 mV (CI 96.1 to 106.6 mV) for Stanslowsky and 93.0 mV (CI 88.9 to 97.1 mV) for Fjodorova protocol, Fig. 7D). In addition, the AP time-to-peak feature shows opposite results for the Ctrl2 and HD72 neurons of the two different protocols (Fig. 7E). Most of the neurons recorded in current-clamp mode presented an AP time-to-peak between 0 and 200 ms. Previous findings revealed a long delay to first spike ranging from 320 to 370 ms characteristic of the striatal MSNS^27,28^. Unfortunately, we found in our preparations 24 out of 309 neurons with an AP time-to-peak longer than 200 ms, so a total of 7.8%, and just one neuron with a time-to-peak longer than 300 ms (dotted lines, Fig. 7E). These results suggest that most of the recorded neurons are non-striatal MSNs but most likely central neurons of other subtypes (Supplementary table S11).

Finally, most neurons generated only a single AP following a depolarization and were therefore characterized as phasic neurons. It appears that neither the differentiation paradigm nor the genotype has a clear effect on the electrical activity of the differentiated central neurons (Fig. 7F and Tables S9 and S10). Overall, these data show here again differences in the excitability and the AP features of the neurons generated by the two protocols, without any obvious effect of the genotype itself.

### Pathologically elevated [Ca^2+^]_i_ does not affect Nav gating or action potential features

**Figure 8:**
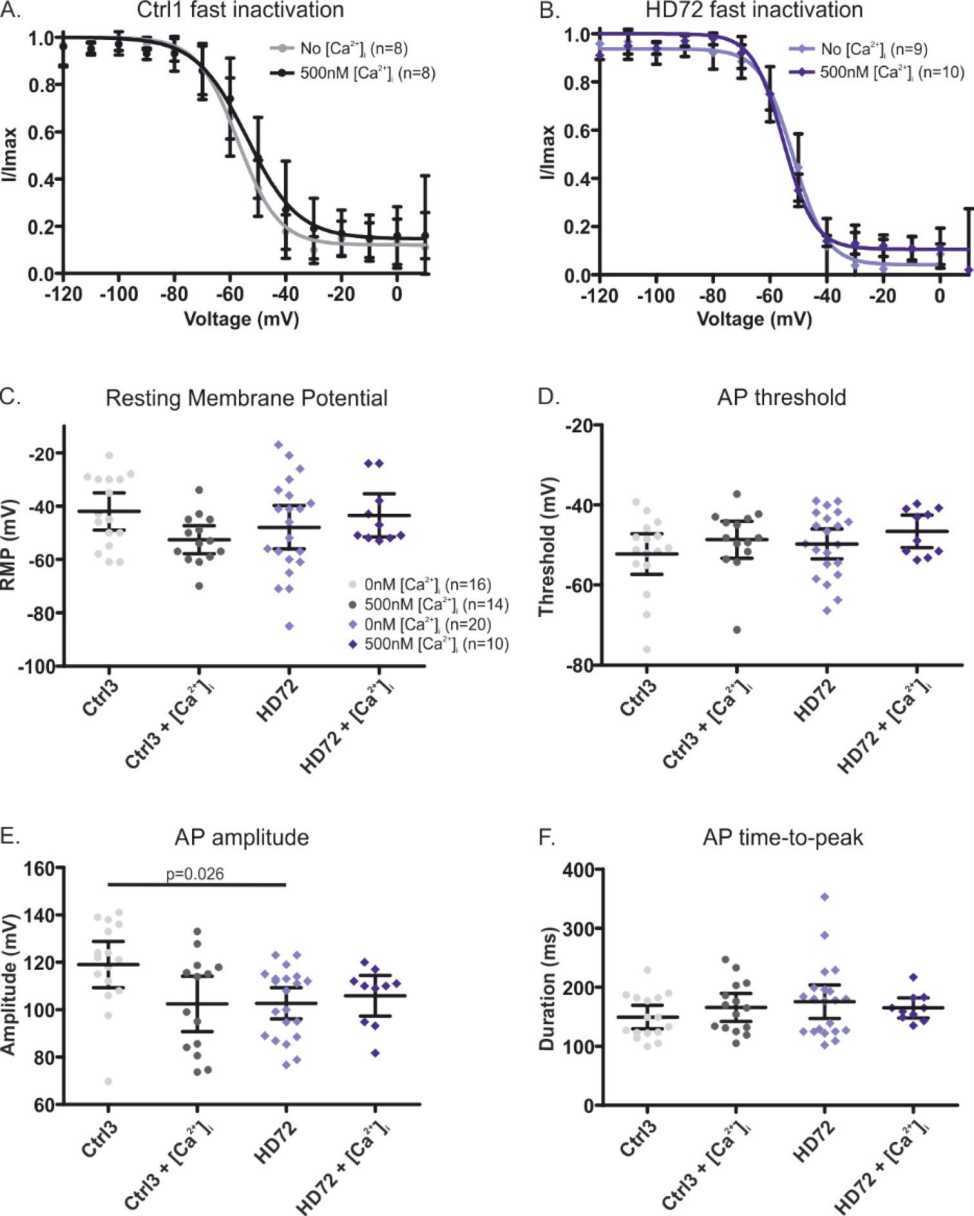
Changing internal Ca^2+^ does not affect steady-state fast inactivation or AP properties of hiPS cell-derived neurons from the Stanslowsky protocol. (**A**, **B**) Steady-state fast inactivation with and without 500 nM [Ca^2+^]_i_ in Ctrl1 (**A**) or in HD72 hiPS cell-induced neurons of the Stanslowsky protocol (**B**) (n = 8–10 neurons). (**C**, **F**) Effect of 500 nM [Ca^2+^]_i_ on AP characteristics in hiPS cell-derived neurons of Ctrl3 and HD72 of the Stanslowsky protocol (n = 9–22 neurons). (**C**) Resting Membrane Potential, (**D**) AP threshold, (**E**) AP amplitude, (**F**) AP time-to-peak. All data shown as mean ± 95% CI. See Supplementary Table S12 for values.

Previous studies established a link between elevated intracellular Ca^2+^ levels and cell apoptosis in a pathological situation where the cells are exposed to stress^29–31^. It is possible that we did not see HD related changes in excitability of the neurons as we did not accurately mimic the pathophysiological environment, such as an elevated [Ca^2+^]_i_. Various rodent models of HD, as a result of Htt overexpression, showed in striatal MSNs increased basal levels of [Ca^2+^]_i_ following glutamate application^31^ and pathogenic cytoplasmic [Ca^2+^]_i_ accumulation^32–34^. Hodgson et al. also showed that [Ca^2+^]_i_ levels were higher in hippocampal neurons of YAC46 mice than in those of YAC18 WT mice (335 ± 70 nM vs 165 ± 12 nM)^35^. We therefore wanted to investigate whether increased [Ca^2+^]_i_ would alter Nav gating and AP firing in hiPS cell-derived HD neurons. First, we tested in transiently transfected HEK293 cells whether gating of hNav1.3, subtype highly expressed in our hiPS cell-derived neurons (Fig. 5), is affected by a high [Ca^2+^]_i_, here 500 nM. Indeed, we found a − 8 mV hyperpolarized shift in hNav1.3 steady-state fast inactivation (Supplementary Fig. S3). To test whether this hyperpolarized shift in steady-state fast inactivation may also be observed in human MSNs expressing multiple Nav isoforms, we performed voltage-clamp recordings with 0 or 500 nM [Ca^2+^]_i_ in Ctr2 and HD72 neurons of the Stanslowsky protocol. However, varying the intracellular [Ca^2+^]_i_ did not affect their gating and steady-state fast inactivation occurred over the same voltage range in both genotypes (ANOVA,* p* > 0.99) (Fig. 8A-B). Again in neurons from the Stanslowsky protocol, we did not find changes in AP firing properties of both Ctrl3 and HD72 neurons under high [Ca^2+^]_i_ (ANOVA, p > 0.99) (Fig. 8C-F). In summary, both Nav gating properties and AP firing of the differentiated neurons remain unchanged under elevated [Ca^2+^]_i._

### Discussion

In this study, we used two protocols for differentiating MSNs from human iPS cells reported in the literature to compare the impact of the HD related CAG-repeats on the neurons’ electrical properties. Both differentiation protocols showed variability and we did not observe an influence of the HD genotype on the electrical functionality of the neurons.

Differentiating hiPS cells into MSNs represents a suitable way to study the neurodegenerative features of HD^13^. We were able to work with three HD cell lines carrying either 72, 109 or 180 CAG repeats (where the HD72 hiPS cell line is the most commonly studied line in the literature^36^). The literature reports a CAG repeat mean of 41 to 45 in patients suffering from HD^37,38^ whereas a CAG repeat length higher than 50 to 75 is associated with juvenile onset^2,39^. However, a study using PCR performed on a small pool of human *post mortem* HD brain tissue revealed 700 to 1,000 CAG repeats in some striatal neurons prior to pathological cell loss^40^. According to the authors, it seems that the CAG repeat expansion occurs earlier in striatal cells than in other regions of the brain. Therefore, we decided to also include a cell line carrying an extreme CAG repeat length to mimic the pathological genetic features of human striatal neurons.

We chose to work with two different previously published differentiation protocols which aim at driving hiPS cells into striatal neurons^14,15^. These two protocols were published to produce similar amounts of GABAergic (around 80%) and DARPP32^+^ (around 40%) neurons^14,26^, percentages higher than in other protocols^4,12,41^.

The two protocols share common small molecules throughout the whole differentiation but vary in specific application of small molecules and in the use of further factors and media. They present three phases of MSN generation: neural induction, regionalization and terminal differentiation^42^. While the Fjodorova protocol works with a monolayer of cells throughout the whole differentiation process, the Stanslowsky protocol mimics the three-germ-layer differentiation (ectoderm, mesoderm, endoderm) by inducing the formation of embryoid bodies (EBs) from hiPS cells. EBs are defined as small aggregates of cells in suspension^14,15^. To our knowledge, the Stanslowsky protocol is the only one reported in the literature using the formation of EBs already during the first day of differentiation.

Six different cell lines have been used throughout this study: Ctrl1, Ctrl2, Ctrl3, HD72, HD109 and HD180. Except for Ctrl3, all cell lines were used for both protocols. HD180 worked well with the Fjodorova protocol but was not successful with the Stanslowsky protocol, which is 20 DIV longer. This could be due to the large CAG expansion which may affect its differentiation potential in this specific paradigm. The HD Consortium showed that the cumulative risk of cell death is significantly higher for the HD180 than for a control cell line (33 CAG repeats) and an overexpression of 134 CAG repeats in this control cell line led to an increase risk of cell death^2,4^. In this context, it is interesting that in our hands, HD180 neurons survived the shorter Fjodorova protocol, suggesting that the critical duration for cell survival lies between DIV35 and 55. Along these lines, the HD109 iPSC-derived neurons were also not affected in our experiments, suggesting that the critical CAG length for accelerated cell death in vitro lies somewhere between 109 and 180 CAG repeats.

Although a generation of around 40% of GABAergic DARPP32^+^ neurons was reported in the protocols^14,15,26^, the amount of DARPP32^+^ neurons was much more variable in our hands, with a range of 5 to 64%, and no DARPP32 expression for the HD109 neurons of the Stanslowsky protocol. Surprisingly, no DARPP32^+^ signal was detected in any hiPS cell-derived neurons of the Fjodorova protocol. This discrepancy is in line with some other previously published protocols that also struggle with the homogeneity and purity of their differentiated neurons, reaching no more than 21%^41^, 14%^12^ or even 5%^4^ DARPP32^+^ MSNs in vitro. The heterogeneity of neuronal populations observed in culture and the low percentage of DARPP32^+^ neurons may be explained by the lack of specificity of some small molecules used in these two protocols, as well as in other published protocols^12,26,42,43^. It is also possible that the tissue of origin and the donor of the cells play an important role for a successful differentiation^44–46^.

We found GABAergic neurons expressing TH and hypothesized that our preparations also contained interneurons. It was previously shown that some TH^+^ neurons do not present the dopaminergic molecular pattern but instead release GABA^47^. In agreement with this study, experiments with mice revealed that TH is found in some striatal GABAergic interneurons which exert a GABAergic inhibition onto striatal MSNs^24^. In addition to this TH/GAD67^+^ neurons, the expression of GABAergic interneuron markers calretinin and somatostatin in two clones of different genotypes reinforced the finding that our preparations contained striatal interneurons as well as MSNs. Somatostatin was exclusively expressed in Tuj1^+^ neurons of the Stanslowsky protocol, here again highlighting discrepancies between the neuronal populations generated by the two protocols (Supplementary Fig. S1 and Table S6). The expression of this GABAergic interneuron marker is relatively high, thus offering the possibility to potentially study GABAergic interneurons with these differentiation protocols (20.3% and 40.9% of the HD72 and Ctrl2 neurons were positive, respectively).

Recent findings revealed that striatal interneurons are involved in HD pathophysiology^48^. However the reported cells were parvalbumin positive, a cell type which we did not detect in our differentiations (Supplementary Table S6).

The RT-qPCR revealed the RNA expression of five sodium channel α isoforms, Nav1.1-Nav1.3, Nav1.6 and Nav1.7 in the iPSC-derived neurons. These results match those obtained from the striatum of WT mice (Supplementary Fig. S3). Surprisingly, except for β3 subunit, none or almost no expression of the β subunits was detected, although their level of expression in mouse striatum is higher than this of the α subunits (Supplementary Fig. S3). Expression of the sodium channel α and β subunits in human central neurons is in accordance with previously published data from the literature. Especially, Nav1.3 is mainly found in the hippocampus and in the striatum^49^ while Nav1.1, 1.2 and 1.6 mRNAs are present in other areas of the CNS^50,51^. It may be surprising to detect Nav1.7 expression in central neurons, since this sodium channel is mainly expressed in nociceptors^52^. But Nav1.7 mRNA was also expressed and regulated in some regions of the CNS, such as the hypothalamus and the olfactory bulb^53^.

We were particularly interested in the level of expression of Navβ4 mRNA between control and HD cell lines. Our in situ hybridization results showed a much higher *Scn4b* expression in the striatum than in the cortex of WT mice (Supplementary Fig. S1). In addition, several studies attested a significant reduction of the striatal β4 subunit expression level in human post mortem tissue as well as in rodent models of HD^7,9^. We wondered whether a β4 mRNA down-regulation may be linked to MSN degeneration and may represent a neurodegenerative marker. Unfortunately, the level of β4 subunit was extremely low in both genotypes and did not allow any statistical comparison test. Accordingly, it was not possible to use the β4 subunit expression as a marker of down-regulation in our study and no conclusion on the role of β4 subunit in the HD pathology can be made from our experiments. Additionally, it might be that hiPS cell-derived central neurons are not old enough to mimic this phenotype. As extending the maturation time of hiPS cell-derived MSNs is not trivial and promotes cell death in vitro (see above), this issue is not easily resolved. Another possibility is that hiPS cell-derived MSNs do present this β4 mRNA down-regulation. But since our hiPS cell-derived cell cultures are poor in DARPP32^+^ neurons, we may have missed a potential difference in β4 expression in the MSNs of each genotype. The absence of any observed effect in our study does not exclude a possible effect in human HD pathogenesis.

The use of hiPS cells is a very valuable technique since it comprises human material carrying the genetic and epigenetic features of both healthy subjects and HD patients^13,26,54^. Unfortunately, our study also points to the limits of such an approach in its current form, which is mainly a low reproducibility of published protocols and a high clone-to-clone variability. This leads to low amounts of neurons of interest in addition to various other neuronal populations in vitro.

Thus, in these conditions, a regulation e.g. of Nav β4 expression, may not be observable, even if it may occur in human HD. This study highlights the gap that still needs to be crossed to recapitulate the pathogenic features of a disease to investigate them properly using hiPS cell derived neurons.

Voltage-clamp data from our hiPS cell-derived neurons revealed an obvious lack of consistency in the Nav function of the neurons generated by the two different protocols. If no change in V_half_ of activation was found either in genotypes or protocols despite a large variability, this was not true concerning the fast inactivation. Surprisingly, we found a more depolarized inactivation in every single clone of the Stanslowsky protocol compared to those of the Fjodorova protocol. Such strong deviations of results from the two differentiation protocols complicate any interpretation about the potential impact of the mutation on ion channel activity or neuronal firing. Moreover, the largest differences were not observed between genotypes but between protocols. This is surprising since the same cell lines were compared in both protocols and we therefore would have expected Nav channels to show the same gating properties. It is possible that the heterogeneous neuronal populations obtained from the two differentiation protocols support different Nav gating behavior, potentially explained by varying Nav channel expression or levels of posttranslational modification. Current-clamp data also revealed counterintuitive results with differences between cell lines found not only in the RMP but also in the AP features. Here again, these data emphasize the large variability among iPSC-derived MSN differentiation protocols established so far^36^ and highlight the fact that data obtained from a single protocol need to be considered with care.

We focused on the AP time-to-peak criterion to assess the potential percentage of MSNs in our cultures, since a long-lasting delay to first spike is characteristic of striatal MSNs. This delay was shown to be around 370 ± 20 ms in mouse striatal MSNs^28^ and as high as 325 ± 20 ms in rat MSNs^27^. The same observation was made in human iPS cell-derived MSNs, with a delay of 200 to 300 ms^26,55^. According to the low number of neurons with a latency above 200 ms to first spike (Fig. 7E), we assumed that most of our recorded neurons were no MSNs, thus confirming our immunostaining results (Supplementary Table S11). In these conditions, it was not possible to determine any potential damaging effect of the mutated huntingtin on the Nav function or the electrical excitability on MSNs, as we most likely did not have many, if any MSNs in our cultures. Thus, additional techniques such as e.g. patch-seq^56^, would only reveal reasonable, interpretable results following an optimization of the existing MSN differentiation protocols to increase the percentage of striatal MSNs generated in vitro.

Previous experiments showed a link between elevated intracellular cytosolic Ca^2+^ levels and cellular apoptosis in dysregulated physiological conditions^29,30^. Particularly, experiments performed with the yeast-artificial chromosome (YAC128) HD mouse model showed a degeneration of MSNs in HD condition following disturbed Ca^2+^ signaling^31,57^. The toxicity of a high intracellular Ca^2+^ concentration on MSNs has not been associated with a potentially impaired neuronal activity but it is known that intracellular Ca^2+^ modulates gating properties or kinetics of voltage-gated sodium channels^58–60^. We decided to use 500 nM as an elevated and potential pathological concentration in our own patch-clamp recordings, as this concentration is reached after repetitive dopamine applications in YAC128 mouse MSNs^57^. However, although we found a hyperpolarized shift of fast inactivation in Nav1.3 expressed in HEK cells (Supplementary Fig. S2), 500 nM [Ca^2+^]_i_ did not influence either Nav channel gating of the hiPS cell-derived neurons or their excitability. Thus, we cannot conclude that pathological [Ca^2+^]_i_ affect HD neurons more strongly than control neurons and it remains unclear whether [Ca^2+^]_i_ regulation really is a hallmark of HD pathophysiology or we just cannot observe it in our model. It should be noted, that again the low amount of DARPP32^+^ neurons in our culture as well as the reduced age of hiPS cell-derived neurons makes it difficult to reliably compare our findings with those of animal models of HD and to make clear-cut conclusions. An optimization of the existing differentiation protocols is essential to reach larger amounts of MSNs in vitro in order to better study the pathogenicity of the mutated *huntingtin* on the function of human MSNs.

### Conclusion

The hiPS cell-derived striatal MSNs represent a powerful tool to investigate genetic disorders such as HD. However, in this study, we have highlighted the challenges in reproducing published data from iPS cell-derived neurons. In addition, large differences were observed in our study between the two differentiation protocols. The two most dramatic drawbacks in MSN differentiation seem to be (1) low number of MSNs compared to other CNS subtypes and (2) low reproducibility between different hiPS cell protocols. This stresses the importance of improving our current differentiation strategies, potentially even taking a substantially different approach such as transdifferentiation^55^ in addition to comparing large datasets ideally from different studies using different protocols. It seems that studying data obtained from only one differentiation protocol dramatically increases the risk of misinterpreting artificial results.

## Availability of materials and data

The datasets generated or analyzed during this study are available from the corresponding author on request. Cell lines can be found in repositories as indicated, permitting conformation with informed consent of donors.

## Supplementary Information

Supplementary Information 1.
