# Supplementary material for: The difficulty to model Huntington’s disease in vitro using striatal medium spiny neurons differentiated from human induced pluripotent stem cells
Source: Sci Rep. 2021 Mar 25;11:6934. doi: 10.1038/s41598-021-85656-x (PMC7994641; doi:10.1038/s41598-021-85656-x)
Supplement: Supplementary file 1 — Supplementary Information 1. [file 41598_2021_85656_MOESM1_ESM.docx]

Supplementary Material and Method

The difficulty to model Huntington’s Disease using human stem cell-derived striatal neurons

Kim Le Cann^1^, Alec Foerster^1^, Corinna Rösseler^1^, Andelain Erickson^1^, Petra Hautvast^1^, Sebastian Giesselmann^2^, Daniel Pensold^3^, Ingo Kurth^2^, Markus Rothermel^4^, Virginia B. Mattis^5^, Geraldine Zimmer-Bensch^3^, Stephan von Hörsten^6^, Bernd Denecke^7^, Tim Clarner^8^, Jannis Meents*^1#^, Angelika Lampert*^1^

^1^Institute of Physiology, RWTH Aachen University Hospital, 52074 Aachen Germany

^2^Intitute of Human Genetic, RWTH Aachen University, 52074 Aachen, Germany

^3^Institute of Zoology, RWTH Aachen University, 52074, Aachen, Germany

^4^Institute für Biology II, Dept. Chemosensation, AG Neuromodulation, 52074, Aachen, Germany

^5^Cedars-Sinai Medical Center, Los Angeles, CA 90048, USA (present address: Fujifilm Cellular Dynamics, Madison, WI, 53711, USA)

^6^Intitute of Virology, Clinical and Molecular Virology, Animal center of preclinical experiments (PETZ), 91054, Erlangen, Germany

^7^IZKF Aachen – RWTH Aachen, 52074 Aachen, Germany

^8^Intitute for Neuroanatomy, MIT 1, 52074 Aachen, Germany

*shared corresponding authors

^#^present address: Multi Channel Systems MCS GmbH, Aspenhaustrasse 21, 72770 Reutlingen, Germany

Correspondence to

Prof. Dr. Angelika Lampert or Dr. Jannis Meents

Institute of Physiology

RWTH Aachen University

Pauwelsstrasse 30

52074 Aachen

Germany

Tel.: +43 2041 80 88811

Email: [alampert@ukaachen.de](mailto:alampert@ukaachen.de); [jmeents@multichannelsystems.com](mailto:jmeents@multichannelsystems.com)

Table of contents

[Origin, reprogramming and CAG repeat number in hiPS cells used in the two differentiation protocols 3](#_Toc58847081)

[Differentiation and passage number of each clone investigated in the two protocols throughout the whole study 4](#_Toc58847082)

[Electrical behavior of hiPS cell-derived neurons of the two protocols at different time points 4](#_Toc58847083)

[Immunostaining results indicate a low amount of MSNs and reveal the presence of striatal interneurons in culture 5](#_Toc58847084)

[Primers for RT-qPCR 6](#_Toc58847085)

[Voltage-clamp and current-clamp data characterize Nav function and neuron excitability in both genotypes and protocols 7](#_Toc58847086)

[Patch-clamp with varying internal Ca^2+^ concentration 9](#_Toc58847087)

[Statistical data 10](#_Toc58847088)

[Mouse supplementary material and method 13](#_Toc58847089)

[References 14](#_Toc58847090)

# Origin, reprogramming and CAG repeat number in hiPS cells used in the two differentiation protocols

Table S1. Origin of the four control hiPS cells and three HD hiPS cell lines reprogrammed from fibroblasts or MSCs used in this study.

| hiPS cells name | Sex, age at sampling | Repository name | Cell origin | References |
| --- | --- | --- | --- | --- |
| Ctrl 1 | F, 3 | - | Fibroblasts | ^1,2^ |
| Ctrl 2 | M, 6 | CS00iCTR21n1 (HD hiPSC Consortium) | Fibroblasts | ^3^ |
| Ctrl 3 | F, 30 | - | Fibroblasts | ^2^ |
| HD72 | F, 20 | GM23225 (Coriell Institute for Medical Research, USA) | Fibroblasts | ^4,5^ |
| HD109 | F, 4 | CS109iHD109n1 (HD hiPS cell Consortium) | Fibroblasts | ^3^ |
| HD180 | M, 6 | CS97iHD180n3 (HD hiPS cell Consortium) | Fibroblasts | ^3^ |

Table S2. Method of reprogramming of the six fibroblast and MSC lines used in this study.

| hiPS cell line | Method of reprogramming | Factors | References |
| --- | --- | --- | --- |
| Ctrl 1 | CytoTune-hiPS 2.0 Sendai Reprogramming Kit (Thermo Fisher Scientific, Schwerte, Germany) | OCT4, KLF4, SOX2, and c-MYC | ^2^ |
| Ctrl 2 | Retroviral transduction (Addgene #27080, #27078, #27077, Cambridge, MA, USA) | OCT4, SOX2, KLF4, L-MYC, LIN28 and p53 shRNA | ^6^ |
| Ctrl 3 | CytoTune-hiPS 2.0 Sendai Reprogramming Kit (Thermo Fisher Scientific, Schwerte, Germany) | OCT4, KLF4, SOX2, and c-MYC | ^2^ |
| HD72 | Retroviral transduction | OCT4, SOX2, KLF4 and MYC | ^4,5^ |
| HD109 | Retroviral transduction (Addgene #27080, #27078, #27077, Cambridge, MA, USA) | OCT4, SOX2, KLF4, L-MYC, LIN28 and p53 shRNA | ^6^ |
| HD180 | Retroviral transduction (Addgene #27080, #27078, #27077, Cambridge, MA, USA) | OCT4, SOX2, KLF4, L-MYC, LIN28 and p53 shRNA | ^6^ |

Table S3. Huntingtin gene CAG repeat number of the six hiPS cell lines. Due to the large expansion of CAG repeats concerning the allele 1 of HD180 hiPS cells, the PCR-assay was not suitable to determine the exact size of the repeat.

| hiPS cell line | Reported CAG repeat number | Measured CAG repeat number |
| --- | --- | --- |
| Ctrl1 | Unknown | Unknown |
| Ctrl2 | Allele 1: 21 ; Allele 2: 18 | Allele 1: 21 ; Allele 2: 18 |
| Ctrl3 | Unknown | Unknown |
| HD72 | Allele 1: 72 ; Allele 2: Unknown | Allele 1: 72 ; Allele 2: 17 |
| HD109 | Allele 1: 109 ; Allele 2: 19 | Allele 1: 118 ; Allele 2: 19 |
| HD180 | Allele 1: 180 ; Allele 2: 18 | Allele 1: larger than 130 ; Allele 2: 18 |

# Differentiation and passage number of each clone investigated in the two protocols throughout the whole study

Table S4. Number of differentiations (=technical replicates) and passage number of each clone.

|  | **Stanslowsky protocol** | | | **Fjodorova protocol** | | |
| --- | --- | --- | --- | --- | --- | --- |
|  | **Passage**  **number** | **Number of differentiations** | | **Passage**  **number** | **Number of differentiations** | |
| **Clones** |  | **RT-qPCR** | **Patch-clamp** |  | **RT-qPCR** | **Patch-clamp** |
| **Ctrl1** | 21, 22, 27 | 2 | 2 | 26, 30 | 1 | 1 |
| **Ctrl2** | 33 (x2), 36 | 2 | 3 | 33 (x2), 41, 37, 48 | 3 | 3 |
| **Ctrl3** | 21, 25, 31, 28 | 1 | 1 | - | - | - |
| **HD72** | 26, 28, 27 (x2) | 3 | 3 | 26, 27, 29, 32, 34, 38, 41 | 1 | 3 |
| **HD109** | 23, 24 | 2 | 2 | 20, 24, 28 | 2 | 1 |
| **HD180** | 18, 18 | 2 | - | 19, 20, 25 | 2 | 2 |

# Electrical behavior of hiPS cell-derived neurons of the two protocols at different time points

Table S5. Percentage of active neurons and of neurons generating 2 or more APs. Top table: Stanslowsky protocol at DIV40 or DIV55. Bottom table: Fjodorova protocol at DIV35.

| Protocol | **Stanslowsky DIV40** | | | | **Stanslowsky DIV55** | | | | |
| --- | --- | --- | --- | --- | --- | --- | --- | --- | --- |
| Cell lines | **Ctrl1** | **Ctrl2** | **Ctrl3** | **HD72** | **Ctrl1** | **Ctrl2** | **Ctrl3** | **HD72** | **HD109** |
| % of cells with 1 AP | 50 | 66.7 | 53.8 | 58.8 | 65.2 | 53.1 | 71.8 | 70.4 | 67.3 |
| % of cells with at least 2 APs | 28.6 | 11.1 | 42.9 | 15.0 | 46.7 | 9.8 | 64.3 | 50 | 18.9 |
| Number of recorded cells | 19 | 18 | 6 | 20 | 60 | 51 | 28 | 38 | 37 |

| Protocol | **Fjodorova DIV35** | | | | |
| --- | --- | --- | --- | --- | --- |
| Cell lines | **Ctrl1** | **Ctrl2** | **HD72** | **HD109** | **HD180** |
| % of cells with 1 AP | 72.3 | 74.5 | 100 | 64.5 | 46.2 |
| % of cells with at least 2 APs | 5.9 | 6.8 | 39.3 | 50 | 6.8 |
| Number of recorded cells | 34 | 73 | 49 | 20 | 73 |

# Immunostaining results indicate a low amount of MSNs and reveal the presence of striatal interneurons in culture


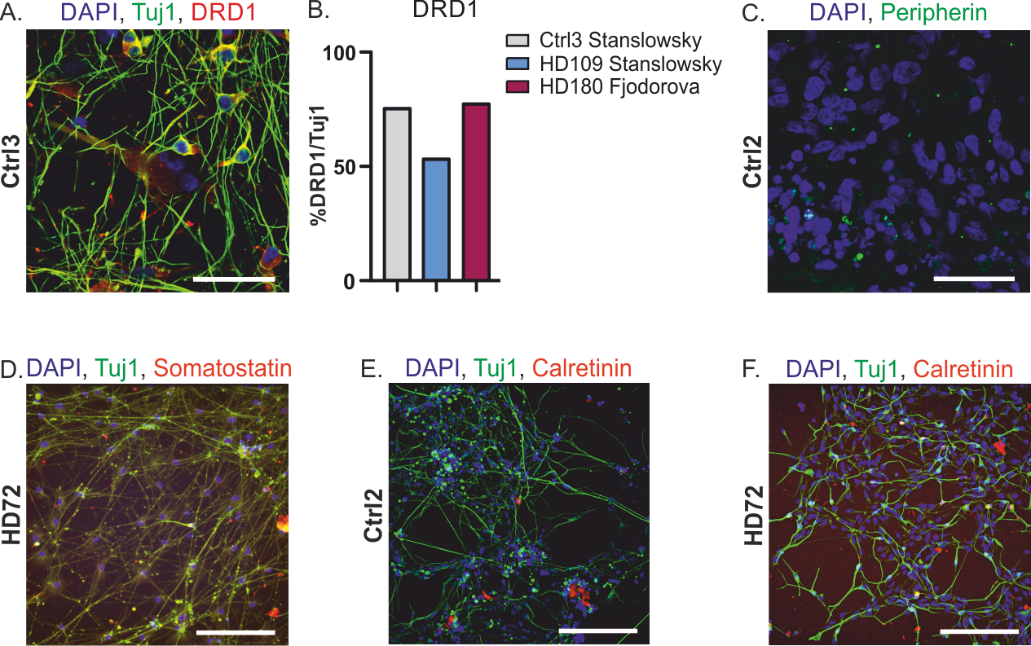


Figure S1. Immunostaining of hiPS cell-derived central neurons. (A) hiPS cell-derived Ctrl3 central neurons using the Stanslowsky protocol, stained with DAPI (blue), Tuj1 (green) and DRD1 (red). (B) Quantification of the amount of DRD1^+^ neurons compared to the number of Tuj1^+^ neurons expressed in hiPS cell-derived central neurons using the Stanslowsky protocol (Ctrl3 and HD109) or the Fjodorova protocol (HD180). Data indicate mean of different areas of interest. (C) hiPS cell-derived Ctrl2 central neurons using the Stanslowsky protocol stained with DAPI (blue) and Peripherin (green). The absence of Peripherin staining confirms a central neuronal identity. Scale bar 50µm. (D-F) Immunostaining of either HD72 or Ctrl2 neurons with interneuron markers. (D) HD72 neurons of the Stanslowsky protocol with DAPI (blue), Tuj1 (green) or Somatostatin (red). (E-F) Ctrl2 neurons of the Stanslowsky protocol (E) and HD72 neurons of the Fjodorova protocol (F) immunostained with DAPI (blue), Tuj1 (green) and Calretinin (red). Scale bar: 200µm.

Table S6. Quantification of co-immunostainings of hiPS cell-derived neurons of each differentiation protocol for the neuronal marker Tuj1, the GABAergic marker GAD67 and TH, the striatal MSN marker DARPP32, as well as the GABAergic interneuron markers calretinin and somatostatin. Data are indicated in percentage, except for the number of regions of interest investigated per coverslip (first line).

| Protocol | Stanslowsky | | | | | Fjodorova | | | | |
| --- | --- | --- | --- | --- | --- | --- | --- | --- | --- | --- |
| Cell line | **Ctr1** | **Ctrl2** | **HD72** | **HD109** | **HD180** | **Ctrl1** | **Ctrl2** | **HD72** | **HD109** | **HD180** |
| Number of regions of interest for each cover slip | 4 - 8 | 3 - 15 | 3 - 22 | 3 - 12 | 4 | 3 - 8 | 2 - 13 | 2 - 15 | 2 - 15 | 3 - 13 |
| Tuj1/DAPI | 9.2 | 33.8 | 26.3 | 40.1 | 13.3 | 14.7 | 25.6 | 27.7 | 31.2 | 19.5 |
| GAD67/DAPI | 18.6 | 22.5 | 34.2 | 36.3 | 0 | 1.1 | 40.2 | 17.3 | 39.3 | 0 |
| DARPP32/GAD67 | 64.4 | 5.2 | 37.2 | 0 | 0 | 0 | 0 | 0 | 0 | 0 |
| GAD67/Tuj1 | - | 74.9 | 79.2 | - | - | - | 82.7 | 74.7 | - | - |
| TH/GAD67 | - | 28.5 | 52.8 | - | - | - | 40.7 | 81.5 | - | - |
| Calretinin/Tuj1 | - | 12.4 | 4.5 | - | - | - | 0 | 9.2 | - | - |
| Somatostatin/Tuj1 | - | 40.9 | 20.3 | - | - | - | 0 | 0 | - | - |
| Parvalbumin | - | 0 | 0 | - | - | - | 0 | 0 | - | - |
| Neuropeptide Y | - | 0 | 0 | - | - | - | 0 | 0 | - | - |
| Neuropeptide VIP | - | 0 | 0 | - | - | - | 0 | 0 | - | - |

# Primers for RT-qPCR

Table S7. Primers used for the RT-qPCR experiments.

| Gene name | Species | Forward primer | Reverse primer |
| --- | --- | --- | --- |
| GAPDH | Human | AGC CAC ATC GCT CAG ACA C | GCC CAA TAC GAC CAA ATC C |
|  | Mouse | GTC ATC CCA GAG CTG AAC GG | ATG CCT GCT TCA CCA CCT TC |
| HPRT | Human | TGA CCT TGA TTT ATT TTG CAT ACC | CGA GCA AGA CGT TCA GTC CT |
|  | Mouse | AGT CCC AGC GTC GTG ATT AG | GCA AGT CTT TCA GTC CTG TCC |
| B2M | Human | GCT CGC GCT ACT CTC TCT TT | TCT CTG CTG GAT GAC GTG AG |
|  | Human | CCT GGT CTT TCT GGT GCT TG | GTT CAG TAT GTT CGG CTT CCC |
| β−actin | Human | CTG GAA CGG TGA AGG TGA CA | AAG GGA CTT CCT GTA ACA ATG CA |
| PSMB4 | Human | CAT TCC GTC CAC TCC CGA TT | CGA ACT TAA CGC CGA GGA CT |
| SCN1A | Human | ATT GAA GGG AAC CGA TTG ACA T | CCA CGG ATG CTC AAC AAA GA |
|  | Mouse | AGG GAA GCA CAG TAG ACA TTG G | AAT CTC TGG ACA CAG CCT TCA G |
| SCN2A | Human | AAG AAT TCA GCA GCG AGT CAG A | GGC GGG AGC TCC AAT ATC AA |
|  | Mouse | ACC TCG CCC TGC TAA CAA G | CGT CCG TTT CCA CCA TCA TG |
| SCN3A | Human | GCT GCA GAG GAA GAC ACG TTA | GCT TCA GTT TTC TTG CTC CAT AA |
|  | Mouse | CCA GAC CAT GTG CCT TAT TGT G | AGC AGC AAG GTT GTC TGA AC |
| SCN4A | Human | CAA GAA GCC TCA GAA GCC AAT T | TGC TTC GTC ACG AGG TCA TAC |
|  | Mouse | GGT GCC ATC TCA GAT GCT ATG | GCA GTT CCA GAT GAG CAC TTT G |
| SCN5A | Human | ATG GTC ATT GGC AAC CTT GTG | AGG GGC TGT GAG GTT GTC T |
|  | Mouse | GTG CCA CCA ATG CCT TGT AC | TCG TGT TGT GCC ATG AAC AC |
| SCN8A | Human | GGA TCC TGA AGG CAG CAA AGA | CCA CAG GGA CCT CTT CTA CTT C |
|  | Mouse | AGA GTC GGA AGA TGG CAT GAG | TTG CTG TTG TGT CGG GAA AG |
| SCN9A | Human | CAC AAT CCC AGC CTC ACA GT | CTG AGG AGC TTG ACC GGT TTA |
|  | Mouse | GAA CAG AAC CAG GCC AAC ATC | AAT CCT GCT CCG CCC TAA AC |
| SCN10A | Human | CTG TCG ATG TCT CGG CAT TC | TGG GCA CTT CTG TTC AGA CTC |
|  | Mouse | TCC CTC ACA AGC CTC ATA GC | ACC AAG GCA TCC ACC ACT AC |
| SCN11A | Human | ACA ACC TGA GCC TGA ACA ACA | CAC TTT GAA CTC TCT GGC TCG T |
|  | Mouse | GTG GAA AGT CCC GAA TGT CAA C | AAT GTC CAG CCA GCC CTT ATA G |
| SCN1B | Human | ACC AAC GCT GAG ACC TTC AC | CCA GCT GCA ACA CCT CAT TC |
|  | Mouse | CGA CTA CGA ATG TCA CGT CTA C | TCT CTG ACA CGA TGG ATG CC |
| SCN2B | Human | CCA CCC TCA ACG TCC TCA ATG | GCG GAA CTG GAG GAA CAT CTC |
|  | Mouse | CCA CGG CAA GAT TTA CCT GC | TGA CCA CCA TCA GCA CCA AG |
| SCN3B | Human | CGA GGG CGG TAA AGA TTT CCT | CCT TGC TGC CAT TCC ACT G |
|  | Mouse | CAC TGA AGA GGC GGG AGA AG | AGC CAC AAG GTG AGG AAG AC |
| SCN4B | Human | TCC TGC TGA TCA AGA AAC TCA TC | AGC TCA CGA GAC ACT CCT T |
|  | Mouse | AGG CAA TAC TCA GGC GAG AT | CCT CCA ACG ACA GGT ACA TG |

# Voltage-clamp and current-clamp data characterize Nav function and neuron excitability in both genotypes and protocols

*Table S8. Voltage-clamp gating properties of hiPS cell-derived central neurons. 95% confidence interval lower and upper values are indicated in brackets.*

| Protocol | Stanslowsky | | | | Fjodorova | | | | |
| --- | --- | --- | --- | --- | --- | --- | --- | --- | --- |
| Voltage-clamp | **Ctrl1** | **Ctrl2** | **HD72** | **HD109** | **Ctrl1** | **Ctrl2** | **HD72** | **HD109** | **HD180** |
| Cell capacitance (pF) | 7.8 (6.9 to 8.8) | 9.7 (6.8 to 12.7) | 9.6 (8.0 to 11.2) | 9.1 (7.3 to 10.9) | 7.2 (6.2 to 8.1) | 8.0 (6.5 to 9.4) | 9.9 (8.9 to 11.0) | 9.4 (6.9 to 11.9) | 7.2 (6.1 to 8.3) |
| Current amplitude (nA) | -2.5 (-3.3 to -1.8) | -1.9 (-2.6 to -1.2) | -2.1 (-2.5 to -1.6) | -3.4 (-4.5 to -2.3) | -1.6 (-2.1 to -1.1) | -2.8 (-3.7 to -1.8) | -2.3 (-3.5 to -1.1) | -2.9 (-3.7 to -2.0) | -2.0 (-2.7 to -1.3) |
| Current density (pA/pF) | -320.6 (-243.2 to -388.0) | -212.6 (-138.5 to -286.7) | -203.8 (-159.7 to -248.0) | -365.4 (-240.9 to -489.8) | -205.6 (-147.5 to -263.6) | -427 (-144.8 to 709.3) | -290.2 (-78.7 to 501.6) | -424 (-290.3 to -557.6) | -197.7 (-149.3 to -246.0) |
| V_1/2_ activation (mV) | -21.2 (-23.0 to -19.4) | -18.7 (-21.3 to -16.1) | -22.2 (-23.9 to -20.6) | -20.8 (-22.5 to -19.2) | -18.5 (-20.0 to -17.0) | -18.7 (-20.1 to -17.4) | -23.1 (-27.7 to -18.5) | -19.0 (-20.4 to -17.7) | -17.3 (-19.6 to -15.0) |
| Slope activation | 6.6 (6.1 to 7.2) | 7.3 (6.5 to 8.2) | 5.7 (4.3 to 7.2) | 6.7 (6.1 to 7.2) | 6.3 (5.8 to 6.8) | 6.3 (5.7 to 6.9) | 5.7 (4.3 to 7.2) | 6.2 (5.7 to 6.6) | 6.6 (6.1 to 7.1) |
| n act. | 26 | 13 | 17 | 14 | 12 | 21 | 5 | 20 | 12 |
| V_1/2_ inactivation (mV) | -52.5 (-54.4 to -50.7) | -57.1 (-59.2 to -55.1) | -54.3 (-55.7 to 53.0) | -61.0 (-63.7 to -58.2) | -65.6 (-68.3 to -62.9) | -66.6 (-67.7 to -65.5) | -58.3 (-61.2 to -55.3) | -66.3 (-67.6 to -65.0) | -60.0 (-61.5 to -58.6) |
| Slope inactivation (mV) | 4.9 (4.3 to 5.5) | 5.6 (5.1 to 6.0) | 5.1 (4.7 to 5.3) | 5.5 (5.0 to 6.0) | 4.8 (4.4 to 5.2) | 5.4 (5.0 to 5.7) | 4.9 (4.6 to 5.1) | 5.5 (5.0 to 5.5) | 5.7 (5.3 to 6.0) |
| n fast inact. | 24 | 23 | 30 | 28 | 16 | 27 | 12 | 26 | 22 |

n act. = number of neurons for activation analysis ; n fast inact. = number of neurons for the fast inactivation analysis

Table S9. Action potential (AP) properties of hiPS cell-derived central neurons. 95% confidence interval lower and upper values are indicated in brackets.

| Protocol | Stanslowsky | | | | Fjodorova | | | | |
| --- | --- | --- | --- | --- | --- | --- | --- | --- | --- |
| Current-clamp | **Ctrl1** | **Ctrl2** | **HD72** | **HD109** | **Ctrl1** | **Ctrl2** | **HD72** | **HD109** | **HD180** |
| n | 27 | 46 | 30 | 37 | 29 | 63 | 58 | 18 | 56 |
| RMP (mV) | -50.1 (-57.1 to -43.1) | -35.91 (-40.2 to -31.7) | -45.1  (-51.6 to -38.7) | -29.3 (-33.5 to -25.2) | -38.1 (-42.9 to -33.3) | -38.2 (-41.9 to -34.4) | -38.8 (-43.3 to -34.2) | -41.1 (-48.5 to -33.7) | -45.6 (-49.2 to -42.0) |
| AP threshold (mV) | -46.5 (-49.3 to -43.7) | -46.0 (-48.5 to -43.5) | -48.0 (-50.9 to -45.0) | -43.8 (-45.5 to -42.1) | -43.8 (-46.6 to -41.0) | -49.8 (-51.5 to -48.1) | -50.0 (-52.1 to -48.0) | -43.6 (-47.6 to -39.7) | -47.7 (-50.2 to -45.3) |
| AP amplitude (mV) | 93.4 (85.0 to 101.9) | 79.3 (75.7 to 82.8) | 101.3 (96.1 to 106.6) | 81.3 (77.2 to 85.3) | 85.2 (81.0 to 89.5) | 87.3 (84.4 to 90.2) | 93.0 (88.9 to 97.1) | 103.1 (93.1 to 113.2) | 87.3 (83.8 to 90.7) |
| AP time-to-peak (ms) | 115.3 (88.2 to 142.3) | 60.6 (43.9 to 77.3) | 146.4 (118.4 to 174.4) | 77.0 (58.1 to 95.8) | 97.0 (72.6 to 121.5) | 96.1 (81.1 to 111.1) | 116.4 (72.6 to 121.5) | 107.4 (76.9 to 138) | 98.9 (81.9 to 115.8) |
| Maximal number of APs | 3.1 (2.0 to 4.3) | 1.6 (1.1 to 2.1) | 6.2 (4.3 to 8.1) | 1.7 (1.0 to 2.4) | 1.4 (1.1 to 1.7) | 2.2 (1.1 to 3.4) | 3.1 (2.3 to 3.9) | 3.5 (1.8 to 5.3) | 1.6 (1.1 to 2.0) |

Table S10. Percentage of neurons firing 1 AP or more than 2 APs, according to Fig. 7F indicating the maximal AP number for each clone

| Maximal AP number – Frequency (%) | | | | | | | | |
| --- | --- | --- | --- | --- | --- | --- | --- | --- |
| Cell line | **Ctrl1** | | **Ctrl2** | | **HD72** | | **HD109** | |
| Protocol | **Stansl.** | **Fjodorova** | **Stansl.** | **Fjodorova** | **Stansl.** | **Fjodorova** | **Stansl.** | **Fjodorova** |
| 1 AP | 53.6 | 76.7 | 85 | 86.7 | 28.1 | 51.7 | 76.9 | 67.6 |
| >2AP | 46.4 | 23.3 | 15 | 13.3 | 71.9 | 48.3 | 23.1 | 32.4 |

*(Stans. = Stanslowsky protocol)*

Table S11. Correlation between GAD67^+^/DARPP32^+^ immunostaining results and percentage of neurons firing APs with a time-to-peak longer than 200ms as an indication of the generation of MSNs by the two protocols.

|  | Stanslowsky | | | | Fjodorova | | | |
| --- | --- | --- | --- | --- | --- | --- | --- | --- |
|  | Ctrl1 | Ctrl2 | HD72 | HD109 | Ctrl1 | Ctrl2 | HD72 | HD109 |
| % DARPP32/GAD67 | 64.4 | 5.2 | 37.2 | 0 | 0 | 0 | 0 | 0 |
| Number of neurons recorded in current-clamp condition | 27 | 46 | 30 | 37 | 29 | 65 | 57 | 18 |
| % neurons with AP time-to-peak>200ms | 14.8 | 2.2 | 20 | 0 | 3.4 | 4.6 | 12.3 | 5.6 |
| Presence of striatal MSNs in culture ? | Yes | Yes | Yes | No | No | No | No | No |

# Patch-clamp with varying internal Ca^2+^ concentration

Table S12. Voltage-clamp gating properties and AP features of hiPS cell-derived central neurons from the Stanslowsky protocol, in presence or in absence of 500nM [Ca^2+^]_i_. 95% confidence interval lower and upper values are indicated in brackets.

| Voltage-clamp data Stanslowsky | | | | |
| --- | --- | --- | --- | --- |
| Cell lines | **Ctrl1** | **Ctrl1 + [Ca^2+^]_i_** | **HD72** | **HD72 + [Ca^2+^]_i_** |
| Cell number | 8 | 8 | 9 | 10 |
| V_1/2_ inactivation (mV) | -53.5 (-59.2 to -47.8) | -47.6 (-56.1 to -39.0) | -52.7 (-56.5 to -48.8) | -53.2 (-54.9 to -51.6) |
| Slope inactivation (mV) | 10.6 (7.3 to 13.8) | 14.6 (7.1 to 22.1) | 7.8 (5.9 to 9.7) | 7.6 (5.9 to 9.4) |
| Current-clamp data Stanslowsky | | | | |
| Cell lines | **Ctrl3** | **Ctrl3 + [Ca^2+^]_i_** | **HD72** | **HD72 + [Ca^2+^]_i_** |
| Cell number | 15 | 14 | 22 | 10 |
| RMP (mV) | -42.0 (-49.0 to -35) | -52.6 (-57.9 to -47.4) | -48.0 (-56.1 to -39.9) | -43.5 (-51.6 to -35.4) |
| AP threshold (mV) | -52.3 (-57.4 to -47.2) | -48.7 (-53.3 to -44.1) | -49.8 (-53.5 to -46.0) | -46.6 (-50.7 to -42.3) |
| AP amplitude (mV) | 119.0 (109.3 to 128.8) | 102.5 (90.8 to 114.2) | 102.7 (96.2 to 109.3) | 105.9 (97.3 to 114.5) |
| AP time-to-peak (ms) | 149.6 (129.7 to 169.4) | 158.7 (139.9 to 180.4) | 175.5 (147.3 to 203.8) | 165.2 (148.1 to 182.3) |


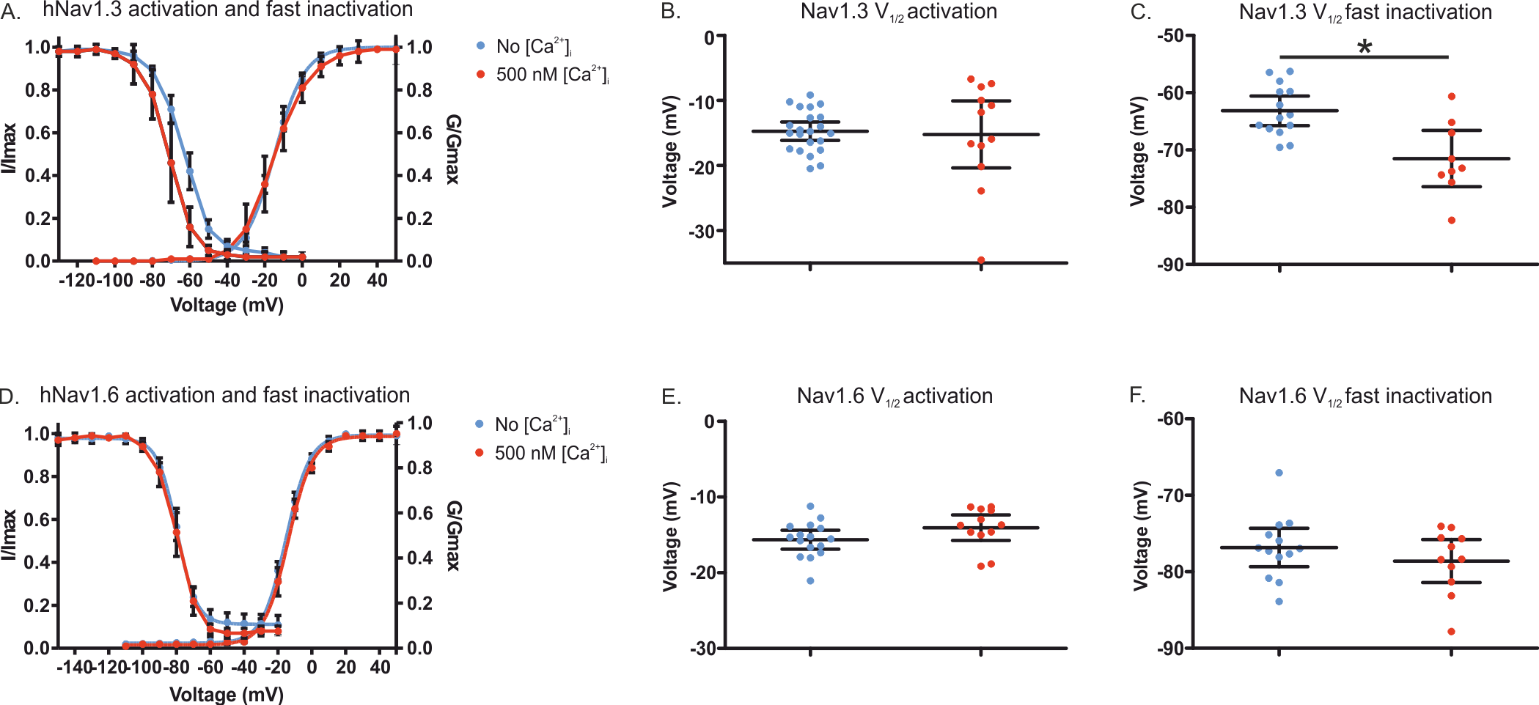


Figure S2. Effects of 500nM [Ca^2+^]_i_ on hNav1.3 and on hNav1.6 expressed in HEK293 cells. (A) Voltage dependence of activation and steady-state fast inactivation of hNav1.3 with and without 500nM intracellular free calcium. (B) hNav1.3 V_half_ of activation without (-14.7mV, 95% CI -16.2 to -13.3mV) and with calcium (-15.2mV, 95% CI -20.4 to -0.1mV) (t(32)=0.25, p=0.80). (C) hNav1.3 V_half_ of fast inactivation without (-63.2mV, 95% CI -65.7 to -60.1mV) and with calcium (-71.5, 95% CI -76.4 to -66.6mV) (t(21)=3.7, p=0.0013). (D) Voltage-dependence of activation and steady-state fast inactivation of hNav1.6 with and without calcium. (E) hNav1.6 V_half_ of activation without (-15.6mV, 95% CI -16.9 to -14.4mV) and with calcium (-14.1mV, 95% CI -15.7 to -12.4mV) (t(26)=1.66, p=0.11). (F) hNav1.6 V_half_ of fast inactivation without (-76.8mV, 95% CI -79.3 to -74.3mV) and with calcium (-78.6mV, 95% CI -81.4 to -75.8mV) (t(22)=1.04, p=0.31). Error bars denote 95% confidence interval.

# Statistical data

The following tables S13A to S13G describe the statistics performed with SPSS for the voltage-clamp data presented in Fig. 6, the current-clamp data presented in Fig.7 and the voltage- and current-clamp data with varying [Ca^2+^]_i_ presented in Fig. 9.

Table S13A. Description of the statistics of V_half_ of activation for the voltage-clamp data depicted in Fig. 6.

| Fig. 6 | V_half_ activation | |
| --- | --- | --- |
| Cell line | **Protocol** | **t-test or U-Mann Whitney** |
| Ctrl1 | **Stanslowsky** | t(36)=1.99, p=0.054 |
|  | **Fjodorova** |  |
| Ctrl2 | **Stanslowsky** | t(32)=0.003, p=0.99 |
|  | **Fjodorova** |  |
| HD72 | **Stanslowsky** | t(21)=1.39, p=0.18 |
|  | **Fjodorova** |  |
| HD109 | **Stanslowsky** | t(40)=1.75, p=0.087 |
|  | **Fjodorova** |  |

Table S13B. Description of the statistics of V_half_ of fast inactivation depicted in Fig. 6.

| Fig. 6 | V_half_ fast inactivation | |
| --- | --- | --- |
| Cell line | **Protocol** | **t-test or U-Mann Whitney** |
| Ctrl2 | **Stanslowsky** | t(38)=8.57, p<0.0001 |
|  | **Fjodorova** |  |
| Ctrl4 | **Stanslowsky** | t(48)=8.76, p<0.0001 |
|  | **Fjodorova** |  |
| HD72 | **Stanslowsky** | t(40)=2.97, p=0.005 |
|  | **Fjodorova** |  |
| HD109 | **Stanslowsky** | t(52)=3.48, p=0.001 |
|  | **Fjodorova** |  |

Table S13C. Description of the statistics of RMP values depicted in Fig. 7.

| Fig. 7 | RMP | |
| --- | --- | --- |
| Cell line | **Protocol** | **t-test or U-Mann Whitney** |
| Ctrl2 | **Stanslowsky** | t(54)=2.95, p=0.0047 |
|  | **Fjodorova** |  |
| Ctrl4 | **Stanslowsky** | t(107)=0.80, p=0.43 |
|  | **Fjodorova** |  |
| HD72 | **Stanslowsky** | t(86)=1.0, p=0.32 |
|  | **Fjodorova** |  |
| HD109 | **Stanslowsky** | U=176.5, p=0.0043 |
|  | **Fjodorova** |  |

Table S13D. Description of the statistics of AP threshold values depicted in Fig. 7.

| Fig. 7 | AP threshold | |
| --- | --- | --- |
| Cell line | **Protocol** | **t-test or U-Mann Whitney** |
| Ctrl2 | **Stanslowsky** | t(54)=1.42, p=0.16 |
|  | **Fjodorova** |  |
| Ctrl4 | **Stanslowsky** | t(109)=2.60, p=0.011 |
|  | **Fjodorova** |  |
| HD72 | **Stanslowsky** | t(85)01.17, p=0.25 |
|  | **Fjodorova** |  |
| HD109 | **Stanslowsky** | t(53)=0.09, p=0.93 |
|  | **Fjodorova** |  |

Table S13E. Description of the statistics of AP amplitude values depicted in Fig. 7.

| Fig. 7 | AP amplitude | |
| --- | --- | --- |
| Cell line | **Protocol** | **t-test or U-Mann Whitney** |
| Ctrl2 | **Stanslowsky** | t(54)=1.81, p=0.075 |
|  | **Fjodorova** |  |
| Ctrl4 | **Stanslowsky** | t(109)=3.56, p=0.0005 |
|  | **Fjodorova** |  |
| HD72 | **Stanslowsky** | t(85)=2.45, p=0.016 |
|  | **Fjodorova** |  |
| HD109 | **Stanslowsky** | t(53)=5.01, p<0.0001 |
|  | **Fjodorova** |  |

Table S13F. Description of the statistics of AP time-to-peak values depicted in Fig. 7.

| **Fig. 7** | **AP time-to-peak** | |
| --- | --- | --- |
| **Cell line** | **Protocol** | **If yes, t-test**  **If not, U-Mann Whitney** |
| **Ctrl2** | **Stanslowsky** | t(53)=1.03, p=0.31 |
|  | **Fjodorova** |  |
| **Ctrl4** | **Stanslowsky** | U=930, p=0.0006 |
|  | **Fjodorova** |  |
| **HD72** | **Stanslowsky** | t(85)=2.0, p=0.049 |
|  | **Fjodorova** |  |
| **HD109** | **Stanslowsky** | t(53)=1.82, p=0.074 |
|  | **Fjodorova** |  |

Table S13G. Description of the statistics of data obtained with varying internal calcium concentrations presented in Fig. 8.

| Fig. 8 | Calcium – Voltage-clamp data – Stanslowsky protocol | | | |
| --- | --- | --- | --- | --- |
| Cell line | | **Condition** | **z-score (skewness value/SE skewness)** | **If z-score<1.96, normal distribution (ANOVA). If not, Kruskall Wallis** |
| Ctrl2 | | No [Ca^2+^]_i_ | 1.45 | F(3, 31)=1.55, p=0.22 |
|  |  | 500nM [Ca^2+^]_i_ | 0.6 |  |
| HD72 | | No [Ca^2+^]_i_ | 0.23 |  |
|  |  | 500nM [Ca^2+^]_i_ | 1.44 |  |
|  | | **Calcium – Current-clamp data – Stanslowsky protocol** | | |
| Ctrl1 RMP | | No [Ca^2+^]_i_ | 0.08 | F(3, 57)=1.67, p=0.18 |
|  |  | 500nM [Ca^2+^]_i_ | 0.34 |  |
| HD72 - RMP | | No [Ca^2+^]_i_ | 0.29 |  |
|  |  | 500nM [Ca^2+^]_i_ | 1.68 |  |
| Ctrl1 - AP threshold | | No [Ca^2+^]_i_ | 2.11 | x2(3)=2.57, p=046 |
|  |  | 500nM [Ca^2+^]_i_ | 2.77 |  |
| HD72 - AP threshold | | No [Ca^2+^]_i_ | 0.97 |  |
|  |  | 500nM [Ca^2+^]_i_ | 0.11 |  |
| Ctrl1 - AP amplitude | | No [Ca^2+^]_i_ | 2.2 | x2(3)=9.69, p=0.021 |
|  |  | 500nM [Ca^2+^]_i_ | 0.16 |  |
| HD72 - AP amplitude | | No [Ca^2+^]_i_ | 0.66 |  |
|  |  | 500nM [Ca^2+^]_i_ | 1.49 |  |
| Ctrl1 - time-to-peak | | No [Ca^2+^]_i_ | 0.99 | x2(3)=2.50, p=0.48 |
|  |  | 500nM [Ca^2+^]_i_ | 1.13 |  |
| HD72 - time-to-peak | | No [Ca^2+^]_i_ | 2.68 |  |
|  |  | 500nM [Ca^2+^]_i_ | 1.57 |  |

# Mouse supplementary material and method

Five WT C57Bl/6 mice of 25 weeks as well as 22 homozygous and heterozygous BACHD mice of 53 to 57 weeks of either sex were used, according to the approval number 54-2532.1-49/12 by local ethical boards of the District Government of Middle Franconia, Bavaria, Germany. Mice were anaesthetized using isoflurane and sacrificed by cervical dislocation. The brain was extracted and 200µm slices were performed using a self-build brain slicer and aluminium razor blades and transferred into cold 10% PBS. The brain slices containing the striatum were visually identified and the striatum was extracted using scalpel and forceps. The pieces of striatum were then mechanically dissociated in a mix of 350µL RA1 Buffer and 3.5µL β-ME. Striatum pieces were stored at -80°C until RNA extraction (see Methods).

*In situ* hybridization (ISH) was performed on two 20-week-old WT males and two heterozygous BACHD mice using the ISH kit (QuantiGene View RNA in situ hybridization tissue assay; Affymetrix-Panomics) according to the manufacturer’s instructions. Briefly, mice were anaesthetized using isoflurane and sacrificed by cervical dislocation. Mice were transcardially perfused with 2% cold paraformaldehyde. After overnight postfixation in the same fixative, brains were dissected, embedded in paraffin and coronary sectioned into five micrometer sections at the levels 215-275 according to the mouse atlas of Sidman *et al*. (<http://www.hms.harvard.edu/research/brain/atlas.html>). Sections were heated at 80°C for 3 min for a deparaffinization process and pre-treated in a boiling solution for 10 min before being treated with a working protease solution (ProteaseQF Kit) for 20 min at 40°C and fixed with 4% formaldehyde for 5 min. The sections were exposed to the probe set solution for 3 hours at 40°C. A pre-amplification hybridization was performed using the PreAmpMixQT for 25 min at 40°C, followed by an amplified hybridization using the AmplifierMixQT for 15 min at 40°C. The hybridization was performed using a mouse DARPP32 RNA probe (*Ppp1r1b*, ViewRNA Type 6), a mouse Nav β4 RNA probe (*Scn4b*, View RNA Type 1 probe) and a mouse Nav1.6 RNA probe (*Scn8a*, ViewRNA Type 1, all Affymetrix eBioscience, Frankfurt, Germany). The pre-warmed probe 6-AP (Fast Blue) against *Scn4b* or *Scn8a* was used first for 15 min at 40°C and then the pre-warmed probe 1-AP (Fast Red) against *Ppp1r1b* was used for 15 min at 40°C. An AP-probe-enhancer-solution was applied at RT for 5min. Slides were then mounted using non-hardening Vectashield mounting medium with Dapi and coverslips. Imaging was done using an LSM 700 laser scanning confocal microscope (Carl Zeiss).


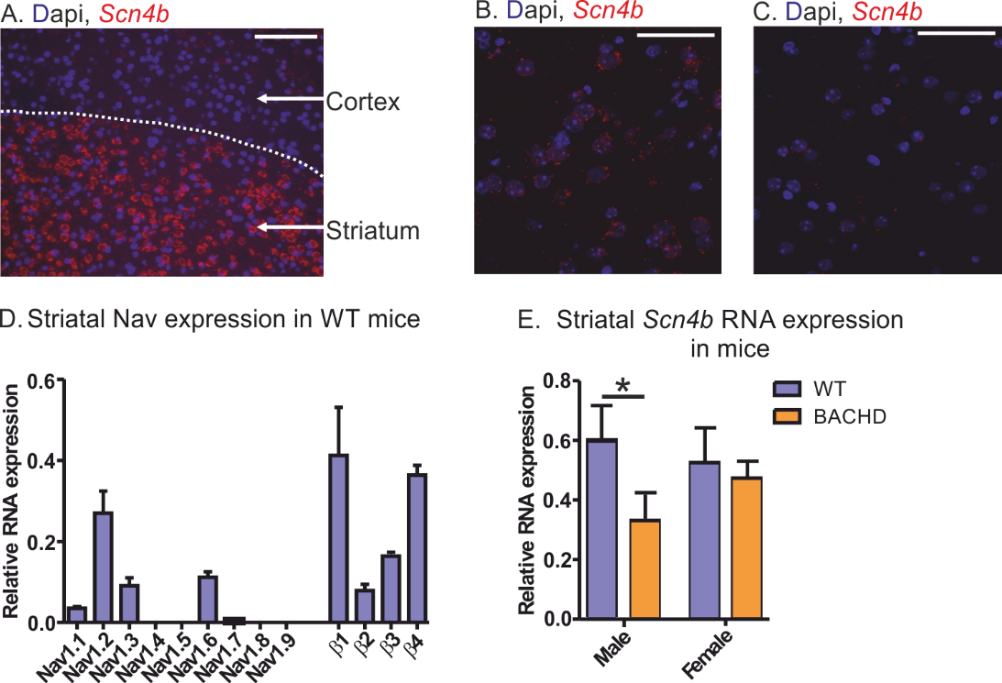


Figure S3. The Nav β4 subunit (Scn4b) is highly expressed in mouse striatum and is down-regulated in the BACHD mouse model of Huntington’s Disease. (A) Expression of Scn4b (red) in the striatum and the cortex of a WT mouse investigated by in situ hybridization. Scale 100µm. Nuclei are stained with DAPI (blue). (B-C) Higher magnification to reveal expression of Scn4b mRNA in the striatum of WT mouse (B) and a BACHD mouse (C). Scale 50μm. (D) RT-qPCR of striatum from 52-week-old mice of both sexes. Expression of genes encoding Nav α and β subunits. Relative mRNA expression was normalized to the geometric mean of the mRNA expression of three housekeeping genes (n=5 mice). (E) Scn4b mRNA expression is reduced in male BACHD mice compared to WT male mice (0.6 (95% CI 0.2 to 1.0) vs 0.33 (95% CI 0.1 to 0.5), n=4-6 animals per group, U=2, p=0.038). Scn4b mRNA expression is not changed in female BACHD mice compared to female WT mice (0.53 (95% CI 0.2 to 0.8) vs 0.47 (95% CI 0.3 to 0.6), n=5 to 7 animals per group, t(10)=0.45, p=0.66). Error bars denote 95% confidence interval.

# References

1. Qin, J. *et al.* Cell fusion enhances mesendodermal differentiation of human induced pluripotent stem cells. *Stem Cells Dev.* **23**, 2875–2882 (2014).

2. Meents, J. *et al.* The role of Nav1.7 in human nociceptors : insights from human induced pluripotent stem cell – derived sensory neurons of erythromelalgia patients. **160**, 1327–1341 (2019).

3. Mattis, V. B. *et al.* Induced Pluripotent Stem Cells from Patients with Huntington’s Disease Show CAG Repeat Expansion Associated Phenotypes. **11**, 264–278 (2012).

4. Cheng, P. H. *et al.* MiR-196a ameliorates phenotypes of huntington disease in cell, transgenic mouse, and induced pluripotent stem cell models. *Am. J. Hum. Genet.* **93**, 306–312 (2013).

5. Tang, Z. *et al.* A dynamic database of microarray-characterized cell lines with various cytogenetic and genomic backgrounds. *G3 Genes, Genomes, Genet.* **3**, 1143–1149 (2013).

6. Mattis, V. B. *et al.* HD iPSC-derived neural progenitors accumulate in culture and are susceptible to BDNF withdrawal due to glutamate toxicity. *Hum. Mol. Genet.* **24**, 3257–3271 (2014).
